# Three-Dimensional Polyglycerol–PEG-Based Hydrogels as a Universal High-Sensitivity Platform for SPR Analysis

**DOI:** 10.1021/acs.analchem.5c00499

**Published:** 2025-03-13

**Authors:** Clemens Krage, Seyma Adigüzel, Boonya Thongrom, Mathias Dimde, Stephan Block, Mohamed Saeed, Maiko Schulze, Florian Junge, Anton Klimek, Katharina Achazi, Roland R. Netz, Uwe Schedler, Rainer Haag

**Affiliations:** † Fachbereich Physik, Freie Universität Berlin, Takustrasse 3, D-14195 Berlin, Germany; ‡ PolyAn GmbH, Schkopauer Ring 6, D-12681 Berlin, Germany

## Abstract

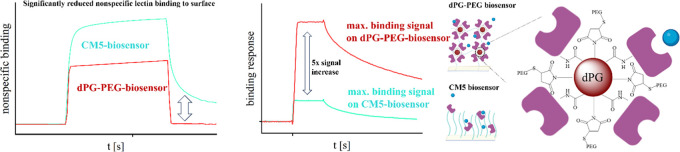

We developed a three-dimensional (3D) polyglycerol–poly(ethylene glycol)-based hydrogel as a new biosensing matrix for affinity analysis by surface plasmon resonance to enable a high loading of ligands for small molecule analysis while lacking a carbohydrate structure to reduce nonspecific binding. The hydrogel was synthesized by cross-linking a polyglycerol functionalized with carboxylate and maleimide groups with a dithiolated poly(ethylene glycol) by thiol-click chemistry. We demonstrated that the hydrogel coating enabled a high immobilization capacity of biomolecules and led to less nonspecific binding. Here, the degree of loading with carbonic anhydrase II and the resulting binding signal of acetazolamide were increased by a factor of 5 compared to standard CMD sensors (CM5), and the loading was comparable to CMD sensors specialized for maximum loading (CM7). This high loading capacity, combined with the reduced nonspecific binding due to the missing carbohydrate structure, presents an innovative matrix for a broad application range of surface plasmon resonance (SPR) experiments since no current commercial SPR biosensor combines these two key features.

## Introduction

Surface plasmon resonance (SPR) has been used as a method to determine affinities for more than 40 years.^1^ In contrast to other methods to analyze the binding behavior, such as microscale thermophoresis (MST) and isothermal titration calorimetry (ITC), SPR can not only determine the binding constant *K*_D_ but also calculate the association constant *k*_a_ and the dissociation constant *k*_d_ by fitting SPR sensorgrams.^2,3^ This is crucial to fully understanding the binding behavior. Additionally, SPR is a label free method and needs significantly less analyte than ITC. The binding constant *K*_D_ in SPR is calculated as follows
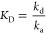
However, surface plasmon resonance still has major drawbacks, restricting its use. The most used SPR sensors consist of a 100 nm carboxymethyldextran (CMD) matrix linked by an alkanethiol layer onto a gold-coated glass slide.^4−9^ This creates a hydrogel where proteins can be immobilized via amine groups of the amino terminus or lysins on the CMD-layer after its ethylcarbodiimide/*N*-hydroxysuccinimide (EDC/NHS)-mediated activation.^7,9,10^ The immobilized protein is defined as the ligand; the other interaction partner, which is then injected over this surface, is defined as the analyte.

The detection is performed by directing light toward the glass-coated side of the gold, creating an electric field along the surface. At a certain angle of incident light and refractive index of the medium on the opposite side, the electric field energy of the photons matches the free electron constellation in the gold, leading to surface plasmon resonance and a detectable minimum of the intensity of the reflected light. The minimum intensity angle shifts according to the refractive index. This refractive index is proportional to the amount of analyte binding to the surface, and the detected signal is proportional to the molecular weight of the analyte.^11−14^ The generated SPR signal is displayed as response units (RU) and is defined by
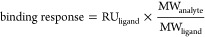


The dependence of the response on the molecular weight (MW) can become an issue when analyzing small molecules as analytes, as the immobilization of ligands is limited by the CMD matrix, thus creating a limited interaction potential with the analyte. Usually, the larger molecule is chosen as the analyte to achieve a high resulting binding signal.

Yet, in some cases, this is not possible, for example, when screening small molecules such as chemically synthesized drugs or when analyzing the binding of enzymes to metal ions. Attaching a linker to the small molecule could create steric hindrance during interaction with the active site and thus falsify the binding constant. The analysis of systems where the ligand is a large molecule (>30 kDa) and the analyte is small (<500 Da) can lead to an insufficient signal of analyte binding due to limited binding sites of the ligand and the small molecular weight of the analyte.^15−17^ The isoelectric point of the immobilized molecule, as well as molecule stability at low pH, may also limit the immobilization capacity of a biomolecule, as covalent immobilization is performed between pH 3.5 and pH 6.^8,18^

To overcome this issue, dendritic polyglycerol (dPG) based polymers have been introduced as matrices to generate more functional groups for biomolecule immobilization.^19,20^ However, the dense packing of the brush macromolecules on the surface tends to lead to a limited accessibility of functional groups, especially if the ligand itself is a large molecule. A significant increase in surface carboxyl groups for coupling could also be reached by other methods; however, the loading capacity of biomolecules could not compete with that of standard CMD biosensors due to the missing spatial separation of the functional groups.^21^ This suggests that a more three-dimensional (3D) setup with less bulky components is necessary to generate an increased availability of carboxyl groups for immobilization. This concept was recently applied in an aptamer selection assay, where a PEG-based hydrogel yielded a three-dimensional porous matrix for aptamer enrichment.^22^ Similarly, agarose–dextran-based gels were shown to enable increased loading capacities in microarrays by creating a three-dimensional matrix, thus lowering the limit of detection of biomolecules.^23^ Further applications of three-dimensional hydrogels include chitosan-based or molecular imprinted poly(*N*-isopropylacrylamide) (PNIPAAm)-based biosensors for biomolecule detection.^24^

A different approach to amplifying the generated signal is to couple the analyte to the nanoparticles. However, these nanoparticles can limit the penetration into the evanescent field if the linker is not carefully selected, and the coupling of a nanoparticle to biomolecules presents a tedious extra step before starting the binding experiment.^25,26^

In addition to the limited loading capacity of a CMD matrix, nonspecific binding of carbohydrate-binding proteins can lead to issues when analyzing lectins due to the carbohydrate structure of the CMD, for instance, heparin-binding proteins.^27−29^ Poly(ethylene glycol) (PEG)-based biosensors were developed to circumvent the nonspecific binding of lectins and to generally reduce nonspecific binding to the gold layer; however, these biosensors have significantly lower immobilization capacities than CMD biosensors due to their single carboxylate group per polymer chain.^29^ This leads to a market gap in biosensors that are able to perform the high-sensitivity analysis of lectin interactions. Furthermore, a more universal biosensor for different types of analysis, such as small molecule analysis and lectin interaction analysis, on the same biosensor would be favorable due to the high cost of individual SPR biosensors.

Another challenge in the design of SPR biosensors is the need to withstand harsh conditions necessary to fully remove the analyte from the biosensor at the end of each cycle. pH changes, as well as high salt concentrations, are often needed to disrupt electrostatic interactions to fully remove the analyte from the surface.^30,31^ Resistance to these conditions without leaching the matrix is crucial for a stable signal and limits the possibilities of the hydrogel components as matrices.

**Scheme 1:**
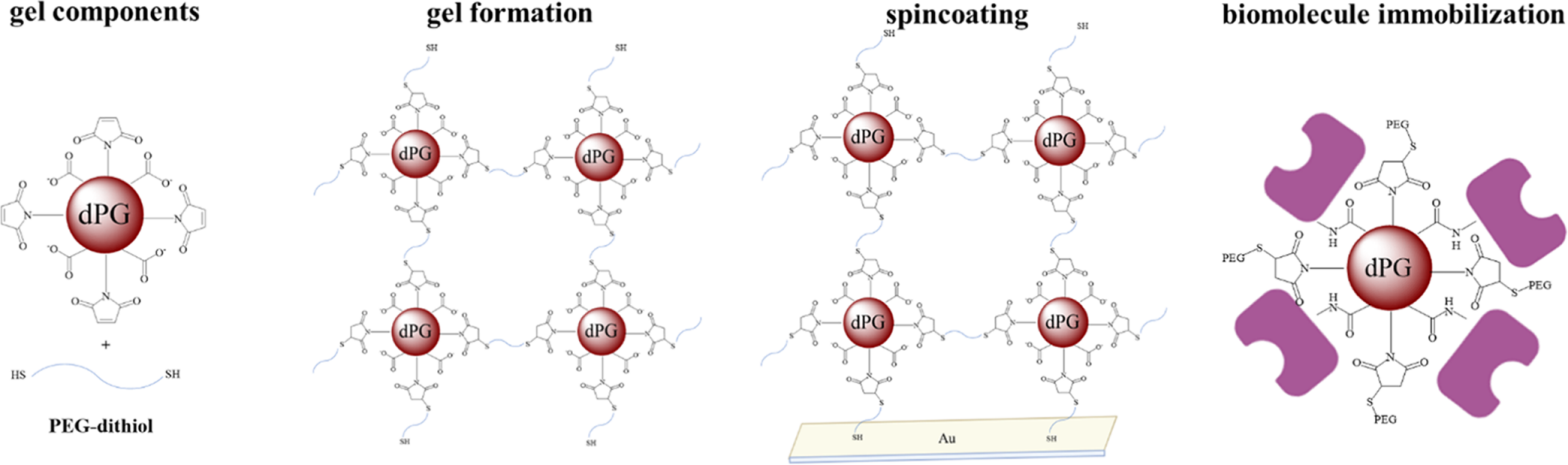
Gel is Formed by Cross-Linking a Maleimide-Containing Hub with PEG-Dithiol by Thiol-Click Chemistry The dendritic polyglycerol-based hub is further functionalized with carboxylate groups to enable covalent biomolecule immobilization via EDC/NHS chemistry. The gel is then spin-coated onto a gold-coated chip by grafting of remaining free thiols.

To overcome the above-mentioned limitations, we created a novel surface-bound hydrogel as a matrix for highly sensitive SPR sensors. This gel consists of carboxylated and maleimide-functionalized dPG for immobilization and a PEG-dithiol-based cross-linker that also acts as a grafting molecule to the surface (Scheme 1). Similar hydrogels based on click-cross-linked PEG and dPG have been severely studied for their rheological characteristics, mesh sizes, and stability.^32,33^ Hydrogels containing dPG as a hub and PEG as a cross-linker have previously been used to capture Enzymes while retaining their full biological activity in a solution-like environment.^33−35^ Additionally, dPG and PEG have been shown to display significant antifouling properties, which prevents nonspecific binding to the gold surface by creating a hydrophilic environment.^19,20,31,36−40^

Micro- and nanogels have previously been developed for SPR analysis to evaluate multivalent protein binding (MPB); however, to our knowledge, no coating could improve the protein loading and resulting signal as well as our dPG–PEG-based hydrogel.^41,42^

In our hydrogel, a significantly increased number of biomolecules can be loaded compared to standard CMD (CM5) sensors due to the spatial separation of the carboxylate groups by the PEG linker and overall more functional groups for immobilization than on a standard dextran chip. This, combined with the reduced nonspecific binding due to the missing carbohydrate structure, makes our dPG–PEG sensors a versatile platform for the SPR analysis of small molecules as well as CMD-binding proteins.

## Materials and Methods

### Synthesis

Dendritic polyglycerol was synthesized as described previously by our group with an average molecular weight of 10 kDa.^43^ dPG was then mesylated and azidated (SI Scheme 1). dPG-azide was then reduced to dPG-amine, yielding 70% total amine groups (SI scheme 2). dPG-amine was then carboxylated with succinic anhydride and conjugated to *N*-succinimidyl-6-maleimidocaproate (SI scheme 3). The detailed synthesis and characterization are described in the Supporting Information (SI Figures 1–10).

### Gel Formation and Spin-Coating Conditions

Several concentrations and ratios of dPGC-mal to PEG-dithiol were tested to generate a hydrogel with a viscosity and gelation time suitable for spin coating (SI Table 1). The conditions in Table 1 generated a hydrogel after 10 min of gelation time. The gold-coated surfaces (SIA kit AU, cytiva) were coated at 600 rpm for 180 s and then dried at 1500 rpm for 120 s. The coated slides were then washed overnight in deionized water.

**Table 1:** Gel Components

c (PEG-dithiol)	V (PEG-dithiol)	M (PEG-dithiol)	*c* (dPGC-mal)	V (dPGC-mal)	M (dPGC-mal)	V (PBS)	c (gel)
10% w	20 μL	6 kDa	10% w	50 μL	20 kDa	30 μL	7% w

### Coating Thickness Measurements

The dry thickness and homogeneity of the gel were analyzed via spectroscopic ellipsometry (SE). We observed that the spin-coating process yielded a stable, homogeneous layer. The thickness of the swollen gel was approximated by atomic force microscopy (AFM) due to the SPR chip being too thin for fixation in the wet state ellipsometry chamber (SI Figures 12–13). Briefly, the coated hydrogel was cut with a scalpel down to the gold layer as a reference point. The thickness of the hydrogel was then determined by calculating the distance from the lowest point to the surface.

### Mesh Size Calculation by Diffusion Analysis of FITC-Labeled Dextran

**Figure 1:**
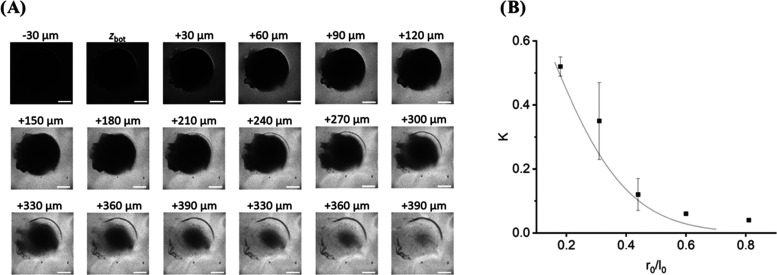
(A) Hydrogel droplet was placed on a glass surface and then incubated with differently sized FITC-dextrans or an FITC-labeled antibody. The diffusion of biomolecules of different molecular weights into the hydrogel was analyzed by calculating the partition coefficient after 30 min incubation time. This image series shows a representative example using a FITC-labeled antibody. Scale bars: 500 μm. (B) The hydrogel mesh size is extracted via a fit of the theoretical prediction for the partition coefficient K depending on the size ratio of the hydrodynamic radius r_0_ of FITC-dextran to the edge length *l*_0_ of PEG linkers in the absence of dextran yielding a mesh size of 8.7 nm.^44^

To confirm the ability of diffusion from small molecules up to molecules of 150 kDa through the matrix, we conducted a diffusion-based confocal laser scanning microscopy (CLSM) assay (Figure 1). In these experiments, the hydrogel has been formed at a glass surface and a solution containing FITC-labeled macromolecules was added to the hydrogel as previously described.^44^ After allowing the system to equilibrate for 30 min, CLSM was used to determine the three-dimensional spatial distribution of the FITC-labeled macromolecules in the sample, providing information on the macromolecule concentration within the hydrogel and in the surrounding bulk solution. These experiments were performed with FITC-labeled dextran molecules of well-defined size (ranging from 4 to 70 kDa corresponding to hydrodynamic radii ranging between 1.4 and 6.0 nm; concentration: 0.2 mg/mL) as well as a FITC-labeled antibody (concentration: 0.05 mg/mL).

The partition coefficient measurement was not repeated for the 40 and 70 kDa dextran as their partition coefficient has a negligible impact on the mesh size calculation.

### SPR Experiments

All experiments were performed on a BiaCore X100 (GE Healthcare) at 25 °C. To determine the optimal loading conditions of the CM5 biosensor, CAII solutions at pH 4.0, 4.5, 5.0, and 5.5 were injected on the CMD biosensor (SI Figure 16). The low pH leads to a net-positive charge of the protein and, thus, electrostatic attraction to the negatively charged CMD.^18^ The steepest injection slopes were reached for pH 4.5 and pH 5.0; however, the following injection of acetazolamide showed no binding at pH 4.5 and a reduced binding signal at pH 5.0 due to protein degradation at this pH. Thus, ab immobilization buffer at pH 5.5 was chosen to investigate the loading capacity. Then, a triplicate concentration series of seven different concentrations were created by 1:2 dilution and injected at an association time of 60 s and a dissociation time of 180 s. Each experiment was performed on three different dPG–PEG or CM5 biosensors and two different CM7 biosensors (SI Figures 17, 19–21, 22–24). The binding constant *K*_D_, as well as the kinetic parameters in Table 5, were obtained by fitting the curves to a 1:1 kinetic fit in the BIAevaluation software (SI Figure 26).

Mouse IgG2b kappa (Invitrogen) was diluted to 25 μg/mL in 10 mM sodium acetate buffer (pH = 5) and injected onto the EDC/NHS-activated preconditioned biosensor (SI Figure 27–28). The remaining active esters were quenched with a 1 M ethanolamine solution. Mouse IgG2bκ was injected in pulses until 300 RU were reached. Triplicate concentration series of five different concentrations of FITC anti-mouse IgG2b (Biolegend clone RMG2b-1) were prepared by 1:1 dilution (5, 2.5, 1.25, 0.625, 0.3125 μg/mL) and injected at an association time of 150 s and a dissociation time of 600 s. All concentrations were injected in triplicates and onto three individual biosensors (SI Figures 29–30).

## Results and Discussion

### Gel Formation and Spin Coating

Our requirements for the gel were a hub molecule with carboxylate groups to preconcentrate and immobilize proteins and a cross-linker that also serves as a spacer and yields a mesh size large enough not to hinder the diffusion and binding behavior of large proteins. A hydrogel consisting of a carboxylate- and maleimide-functionalized dPG was chosen as a hub molecule, and PEG-dithiol as a cross-linker as well as a grafting molecule to attach to the gold surface. The Michael addition of the thiol groups of the cross-linker to the maleimide groups of the hub leads to a fast gelation and is free of any byproducts. This leads to a three-dimensional network, where the mesh size is determined by the length and amount of the PEG-dithiol linker. First, the hydroxyl groups of dPG were converted to amines before coupling the carboxylate and maleimide groups via amide coupling to obtain a maleimide-containing dendritic poly(glycerol carboxylate) (dPGC-mal).

An amide-based coupling was chosen to avoid leaching of the hydrogel, which has been observed before for similar hydrogels consisting of dPG–PEG Gels cross-linked by acrylate chemistry.^45^ Acidic or basic regeneration conditions needed in SPR for surface regeneration would accelerate this process.

The selected gel conditions (Table 1) yielded a thickness of 8 nm in a dry state (*n* = 3). A layer thickness of 58 nm (*n* = 2) was detected in the wet state. This leads to a relatively high swelling ratio of 7.25, which is beneficial for the diffusion of biomolecules through the gel. A similar swelling ratio was observed when coating gold-coated silicon wafers with the gel and consecutive wet state SE measurements (Table 2).

**Table 2:** Layer Thicknesses on SPR Sensors

	dry	swollen	swelling ratio
thickness SPR sensor [nm]	8 ± 2 (SE)	58 ± 3 (AFM)	7.25
thickness gold wafer [nm]	16 ± 3 (SE)	107 ± 3 (SE)	6.54

### Gel Morphology Analysis

The gel homogeneity after spin coating was first confirmed by SEM (SI Figure 14A). To confirm a homogeneous distribution of protein after immobilization, a fluorescent-labeled antibody (FITC anti-mouse IgG2b) was covalently immobilized in the hydrogel. CLSM confirmed a homogeneous distribution of fluorescence over the chip (SI Figure 14C). Negative control was performed by analyzing a nontreated dPG–PEG biosensor with FITC anti-mouse IgG2b to rule out the fluorescence signal resulting from artifacts.

### Mesh Size Calculation by Diffusion Analysis of FITC-Labeled Dextran

Figure 1A shows the cross-sectional distribution of the antibody concentration (indicated by gray color coding) at different *z*-levels of the sample, measured relative to the z-level of the glass bottom hosting the hydrogel. Due to steric repulsion, only a fraction of the FITC-labeled antibody or dextran is able to enter the hydrogel, causing the hydrogel to appear as a dark, circular structure. The intensity contrast between the dark (hydrogel) and bright areas (bulk phase) allows quantification of the fraction of FITC-labeled macromolecules that entered the hydrogel (i.e., the partition coefficient of the macromolecule) (SI Figure 15).

Table 3 shows that all dextrans showed diffusion into the gel, confirming the molecules’ ability to flow through the dPG–PEG Matrix instead of binding events during SPR analysis just taking place on the gel surface. Interestingly, the anti-mouse IgG2b antibody showed significantly better diffusion into the gel with a partition coefficient of 0.19 ± 0.08 than the 70 kDa dextran despite its molecular weight being approximately twice as large (150 kDa). This may result from the different morphology of the dextran and the antibody. While the dextran is a linear molecule, the antibody is folded, leading to a reduced hydrodynamic radius. Electrostatic interactions of the negatively charged hydrogel with the dextrans or antibody due to the labeling with negatively charged Fluorescein might also contribute to the faster dissociation of the antibody into the gel. These electrostatic interactions have been observed before on CMD biosensors, such as negatively charged ligands like nucleic acids not being able to be immobilized in the matrix due to electrostatic repulsion of the negative charge. This issue can be addressed by in situ reversed-charge preconcentration.^46^ The mesh size was determined by using a previously developed theoretical model predicting the partition coefficient for a certain mesh size and Stokes radius of dextrans, taking into consideration the free volume exclusion and flexibility of the polymers (Figure 1B). This yielded an average mesh size of 8.7 nm. However, this does not mean that larger molecules cannot travel through the gel. Previous studies on the diffusion properties of molecules in PG–PEG hydrogels determined that these hydrogels have a large pore size distribution, and molecules in the matrix predominantly travel through the larger pores.^44^

**Table 3:** Partition Coefficients of Different-Sized FITC-Labeled Dextrans

dextran MW (kDa)	Stokes radius *r*_0_ (nm)	partition coefficient (*c*_gel_/*c*_bulk_)
4	1.4	0.52 ± 0.03
10	2.3	0.35 ± 0.12
20	3.3	0.12 ± 0.05
40	4.5	0.06
70	6.0	0.04

### SPR Assay—Small Molecules

**Figure 2:**
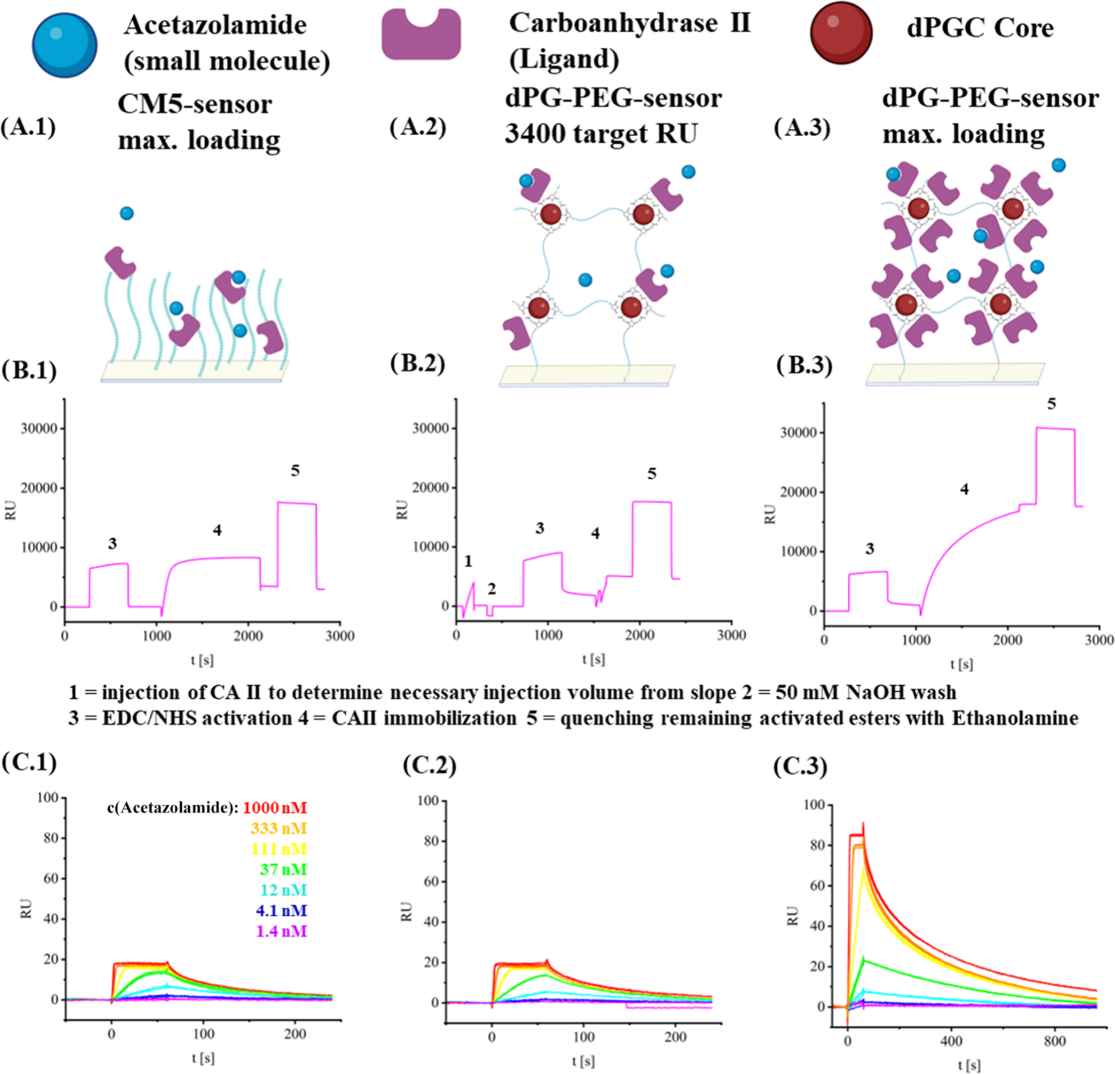
(A) Scheme of a fully loaded CM5 (A.1), a partially loaded dPG–PEG biosensor (A.2), and a fully loaded dPG–PEG biosensor (A.3). (B) Representative immobilization sensorgrams of CAII on different biosensors. The CM5 (B.1) biosensor’s maximum loading capacity was reached at 3400 RU, while on average 17 100 RU could be immobilized on a dPG–PEG biosensor (B3). In B.2, specifically, 3400 RU were immobilized on a dPG–PEG biosensor to compare the resulting binding sensorgrams to a CM5 biosensor using the target immobilization function in the BiaCore X100 Control software. All measurements were performed on 3 different sensors of each type (SI Figures 17,19–20). (C) The resulting representative binding sensorgram of acetazolamide to CAII-immobilized biosensors. A significant increase in the binding signal was reached on maximum loaded dPG–PEG biosensors (C.3) compared to CM5 biosensors (C.1) or only partially loaded biosensors (C.2). All measurements were performed on 3 different sensors of each type (SI Figures 23–25).

To demonstrate the superiority of our system to conventional CMD biosensors, the loading capacity of our gel was compared to that of a commercial CM5 biosensor (GE Healthcare) on a BiaCore X100 (GE Healthcare) and to CM7 biosensors (GE Healthcare), which are optimized for maximum loading. We investigated the binding of the small molecule acetazolamide to the enzyme carbonic anhydrase II (CAII) (Figure 2A.1, 2A.2, 2A.3).

This binding system has been extensively investigated before, and the resulting binding constants have been validated previously by ITC to rule out artifacts resulting from enzyme immobilization by others.^47^ The loading capacity of the protein and the maximum binding response of the small molecule were chosen as critical performance parameters. Additionally, the obtained binding constant *K*_D_ and the kinetic parameters were compared to those obtained on a CM5 biosensor as a control.

Figure 2B.1 shows that the maximum loading capacity of the EDC/NHS-activated CM5 biosensor was reached at 3400 RU. Performing the same procedure on a PEG biosensor led to 345 RU of immobilized protein (SI Figure 18). Pulses of the same enzyme solution were then injected onto an EDC/NHS-activated dPG–PEG biosensor until 3400 RU was reached here as well (Figure 2B.2). Only 22 μL of protein solution were needed to reach the 3400 RU while 130 μL of protein solution were necessary to immobilize the same RU on the CM5 biosensor. Injection of pulses of protein solution instead of a continuous injection on a CM5 biosensor led to only half as much immobilized protein even after 130 μL of injected protein solution (SI Figure 22). Then, seven different concentrations of acetazolamide were injected over both types of sensors, yielding consistent binding constants and the expected RU signals. To determine the maximum loading capacity of the dPG–PEG biosensor, CAII was injected under the same conditions as on the CM5 biosensor in Figure 2B.3. This led to an average 5-fold increase in loading capacity compared to CM5 biosensors and a slightly improved loading capacity (17 100 RU vs 16 400 RU) compared to the specialized CM7 biosensors (SI Figure 21). The following analyte injection in Figure 2C.3 led to the expected increase in the signal proportional to the amount of immobilized ligand. A longer dissociation time was chosen in this experimental setup, as the necessary time for full dissociation is increased. This also explains the different morphology of the analyte response curves, as an increased association time would be necessary to reach a binding equilibrium at the lower concentrations due to higher availability of overall binding sites than in Figure 2C.1,2C.2, where significantly less CAII is immobilized. The resulting kinetic data, however, still confer to that of the binding experiment on a CM5 biosensor, which was used as a control to determine the binding constants *K*_D_, *k*_d_, and *k*_a_ (Table 4).

**Table 4:** Comparison of Binding Constant and Loading Capacity

chip type	*K*_D_ [nM]	10^6^*k*_a_ [1/Ms]	*k*_d_ [1/s]	loading capacity CAII in 1000 RU
CM5 sensor maximum loading (CMD)	13.8 ± 1.7	4.7 ± 2.8	0.06 ± 0.03	3.4 ± 0.3
dPG–PEG sensor 3400 RU loading	12.8 ± 2.5	2.7 ± 1.9	0.04 ± 0.03	
dPG–PEG sensor maximum loading	12.8 ± 2.6	3.4 ± 2.3	0.04 ± 0.02	17.1 ± 4.1

### SPR Assay—Large Molecules

**Figure 3:**
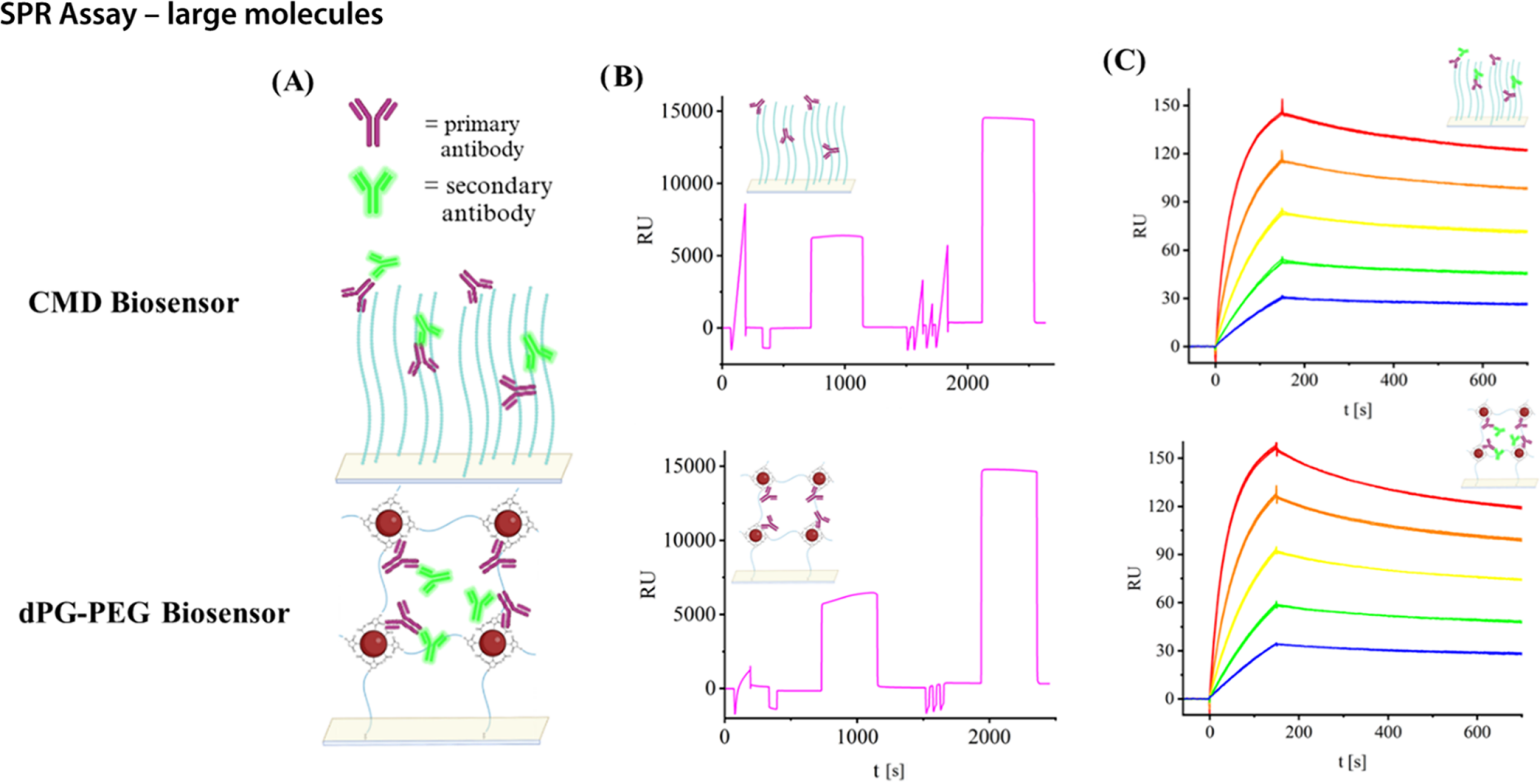
(A) Scheme of the interaction on a CM5 Sensor (left) and a dPG–PEG sensor (right). (B) Primary antibody mouse IgG2bκ was injected in pulses until 300 RU were reached. All immobilization experiments were performed on three individual biosensors (SI Figure 27). (C) Binding response of secondary antibody FITC rat anti-mouse IgG2b to the primary antibody. C (secondary antibody): 5 μg/mL (red), 2.5 μg/mL (orange), 1.25 μg/mL (yellow), 0.625 μg/mL (green), and 0.3125 μg/mL (blue).

To investigate whether large molecules up to 150 kDa can freely diffuse through the gel and be analyzed in this matrix, the interaction of two IgG antibodies was analyzed (Figure 3A). In this setup, a primary antibody (Mouse IgG2b) was immobilized (Figure 3B). Anti-mouse IgG was chosen as the analyte and then injected at five different concentrations to obtain binding curves to compare the resulting binding constants between CM5 and dPG–PEG biosensor (Figure 3C). The comparable binding constants in Table 5 determined by fitting in the BIAevaluation software (SI Figure 31) confirm that the hydrogel does not have diffusion-limiting effects (mass transport limitation), leading to false binding constants when analyzing large molecules such as IgG antibodies in an SPR setup. Thus, the dPG–PEG-based sensors are suitable for small molecule as well as large molecule analysis.

**Table 5:** Comparison of the Resulting *K*_D_ Values

chip type	10^6^*k*_a_ [1/Ms]	10^–4^*k*_d_ [1/s]	*K*_D_ [pM]
CM5 sensor	1.2 ± 0.1	3.9 ± 0.7	307 ± 87
dPG–PEG sensor	1.1 ± 0.0	3.4 ± 0.8	310 ± 40

### Investigation of Nonspecific Binding to the Surface

**Figure 4:**
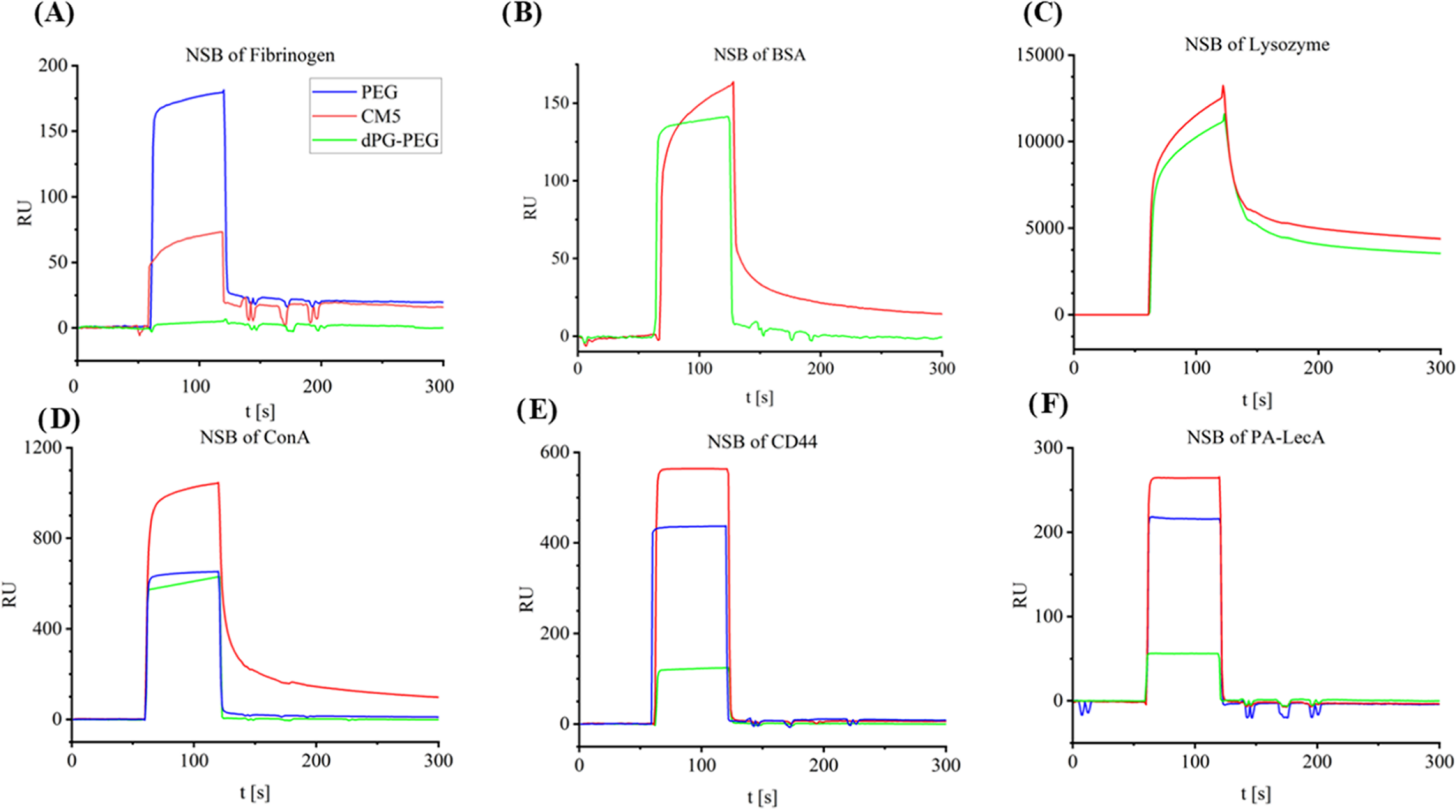
One mg/mL Fibrinogen (A), BSA (B), lysozyme (C), Con A (D), 0.1 mg/mL CD44 (E), and 1 mg/mL PA-LecA (F) were injected over CM5 and dPG–PEG biosensors for 60 s to investigate the nonspecific binding. The nonspecific binding was also compared to PEG biosensors in (A) and (D)–(E).

The selectivity of the chip was investigated by injecting bovine serum albumin (BSA), Lysozyme, Fibrinogen, Concanavalin A (Con A), cluster of differentiation protein 44 (CD44) and*Pseudomonas aeruginosa* Lectin A (PA-LecA) over different types of biosensors (Figure 4).

BSA and Lysozyme showed slightly reduced nonspecific binding on the dPG–PEG biosensor compared with CM5 biosensors. This effect is likely due to the more hydrophilic structure of our gel components (PEG and dPG) than that of the CMD. This has been observed previously when coating gold surfaces with a monolayer of dPG.^20^ The generally high adsorption of lysozyme can be explained by the electrostatic interaction of positive patches on the lysozyme surface with the negatively charged carboxylates of the CMD or dPG–PEG.^48^ Interestingly, a significant reduction in the binding of fibrinogen to the surface was also observed.

For all lectins (Con A, CD44, and PA-LecA), a significant reduction in nonspecific binding to the surface was observed compared to that of CM5 and PEG biosensors. Most notably, Con A sticks to the CMD surface after the injection (Figure 4D), most likely due to the carbohydrate structure of the CMD, as the native binding partners of Con A are glucose and mannose. On the dPG–PEG biosensors, however, the signal drops right back to baseline after the injection of Con A is finished. This makes the dPG–PEG biosensor a better platform for the investigation of interactions of lectins such as Con A.

## Conclusions

Our study shows that dPG–PEG biosensors enable an average loading of the matrix five times larger than on a standard CM5 biosensor, thus being a great tool for small molecule analysis that cannot be analyzed by conventional CM5 biosensors. The loading capacity is also slightly better than that of a CM7 sensor, which is a CMD sensor optimized for maximum loading. We also confirmed that the dPG–PEG biosensors yield accurate binding constants by comparing them with those obtained on a CMD biosensor. By this, we could rule out possible diffusion-limiting effects of the matrix that would lead to falsified kinetic constants. Additionally, the dPG–PEG biosensors need significantly less protein for immobilization to reach the same response level as that of CM5 biosensors. This can be advantageous when working with costly biomolecules. We then demonstrated that interaction analysis of two large molecules of approximately 150 kDa is possible with the dPG–PEG-based biosensor, as it yields the same data as a CMD biosensor. Moreover, we determined that dextrans up to 70 kDa and IgG antibodies (150 kDa) penetrate the hydrogel. While FITC anti-mouse IgG2b showed only a partition coefficient of 0.19 in the diffusion experiment, the resulting binding constant when analyzing this molecule was still the same as when analyzed via the CMD biosensor. This proves that the diffusion behavior of IgG2b in the SPR setup is not mass transport controlled, and the calculated average mesh size of 8.7 nm does not hinder the binding behavior of molecules up to 150 kDa. This likely results from the drastically reduced thickness of the gel on the SPR Chip of 58 nm compared to approximately 1 mm in the CLSM experiment.

dPG–PEG biosensors also present a great alternative to CMD biosensors for lectin analysis, as they lack the lectin-binding carbohydrate structure. The nonspecific binding to the surface was investigated by injecting BSA, lysozyme, fibrinogen, and a series of carbohydrate-binding proteins over the surface. Here, less nonspecific binding was observed on the dPG–PEG biosensor than on the CMD biosensor. The combination of the high immobilization capacity with reduced nonspecific binding of CMD-binding proteins makes our biosensors a great universal alternative to CMD- or PEG-based biosensors. No current commercial SPR sensor combines these two key features since PEG biosensors lack the high loading capacity (SI Figure 18), and CMD (CM5, CM7) biosensors lead to high nonspecific binding of lectins (Figure 4) and other proteins.

Finally, the hydrogel’s ability to withstand harsh regeneration conditions such as acids, bases, or highly concentrated salt solutions was confirmed, as these are often necessary to fully remove the analyte from the surface (SI Figure 32). The simple synthetic conditions of the gel components enable further introduction of functional groups for various common immobilization chemistries, such as nickel-nitrilotriacetic acid (NTA), Streptavidin–biotin, and others. Additionally, introductions of azide or dibenzocyclooctyne moieties into dPG-based systems have been performed previously and could enable strain-promoted azide–alkyne coupling for biorthogonal immobilizations of nonrobust proteins or polymers, making the gel a versatile platform for further applications.^33,49^

## Supporting Information Available

Synthesis; surface analysis; and binding experiments (PDF)
