# Supplementary material for: Three-Dimensional Polyglycerol–PEG-Based Hydrogels as a Universal High-Sensitivity Platform for SPR Analysis
Source: Anal Chem. 2025 Mar 13;97(11):6329–37. doi: 10.1021/acs.analchem.5c00499 (PMC11948182; doi:10.1021/acs.analchem.5c00499)
Supplement: Supplementary file 1 — ac5c00499_si_001.pdf [file ac5c00499_si_001.pdf]

Supporting Information for

Three-dimensional Polyglycerol-PEG-based hydrogels as a universal high-sensitivity platform for SPR Analysis

‡†Clemens Krage, ‡†Seyma Adigüzel, †Boonya Thongrom, †Mathias Dimde, †Stephan Block †Mohamed Saeed, §Maiko Schulze, †Florian Junge ϛAnton Klimek, †Katharina Achazi, ϛRoland R. Netz, §Uwe Schedler, †Rainer Haag.

†Institut für Chemie und Biochemie, Freie Universität Berlin, Takustrasse 3, D-14195 Berlin, Germany
§PolyAn GmbH, Schkopauer Ring 6, D-12681 Berlin, Germany
ϛFachbereich Physik, Freie Universität Berlin, D-14195 Berlin, Germany

Corresponding authors:
Rainer Haag – Department of Biology, Chemistry, Pharmacy, Freie Universität Berlin, E-Mail: haag@chemie.fu-berlin.de
Uwe Schedler – PolyAn GmbH, E-Mail: u.schedler@polyan.de

Contents

Chemicals and Synthesis 2
dPG-Azide 2
dPG-Amine 5
dPG-C-mal 7
PEG dithiol 8
$PEG(OMs)_2$ 8
PEG dithiol 9
NMR of formed gel 10
Surface activation, Ellipsometry and AFM measurements 11
Goniometer measurements 11
Surface activation of the SPR biosensors 11
Coating of the SPR biosensors 11
Gel thickness determination by spectroscopic ellipsometry 11
Wet state gel thickness determination by Atomic force microscopy 12
Surface morphology analysis 13
Partition coefficient determination by diffusion analysis of FITC-labeled Dextran 14
SPR Measurements for biosensor validation 15
Preconcentration scouting: 15
Immobilization 15
SPR Assays- IgG Interactions 18
Immobilization 18
Binding experiments on 300 RU immobilized Sensors 20
Screening of regeneration conditions: 20
SI References 21

Chemicals and Synthesis

All chemicals were purchased from Merck KGaA, Darmstadt, Germany and/or its affiliates and used without any further purification, unless otherwise stated. Diethyl ether (100%), DCM (100%) were purchased from VWR chemicals. *N, N*-Dimethylformamide (99.8%), KOH (pellets), NaN_3 (99%), MeOH (99.9%), THF(99.8%) and Chloroform were purchased from Thermo Fisher Scientific. Triphenylphosphine (99%) was purchased from Roth.

DPG

The average molecular weight of 10 kDa of dPG was prepared as previously reported with the improved method in our group.[1-4]

dPG-Azide

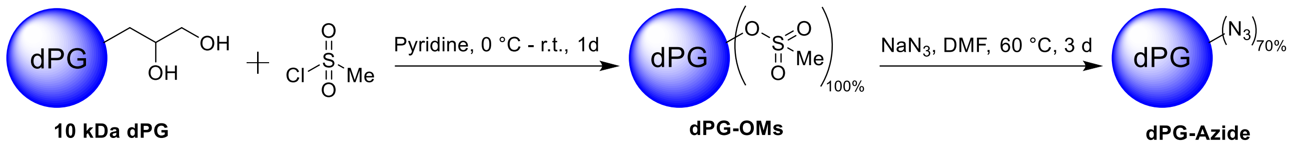

SI Scheme 1: Synthesis of dPG-Azide

Dried 10 kDa dendritic polyglycerol (dPG, 1 eq., 0.5 mmol, 5 g) was dissolved in Pyridine (50 mL) and the reaction flask was cooled down by ice bath. Methanesulfonyl chloride (MsCl, 142 eq., 71 mmol, 5.5 mL) was then added dropwise and the mixture was stirred for 1 day. The precipitate byproduct was filtered off and the filtrate was collected and concentrated, resulting in dPG-OMs product (SI Figure 2). ^{1}H NMR (500 MHz, Pyridine-d5, δ (ppm)): 3.51 – 3.55 (3H, broad s), 3.78 – 5.52 (m, backbone repeating units). The number of Mesylate functional groups (OMs) was calculated by NMR end-group analysis. Each Glycidol repeating unit contains, in theory five protons. So if all OH groups were transformed to mesylate groups (OMs), the number of mesylate protons would be 3. Hence the NMR spectrum in SI Figure 2 showed that dPG was functionalized with approximately 100% mesylate groups (by the ratio of 5:3.03 protons). Glycidol (74 g/mol) contains, in general, 1 free primary OH group. So 1 molecule of 10 kDa dPG should contain approximately 135 free OH groups (10000/74) which is counted as 100% functional group. This number of OH groups was used as equivalent (eq.) in the calculation of the synthetic functionalization. The result of 100% mesylate functional groups on 10 kDa dPG would contain circa 135 mesylate groups.

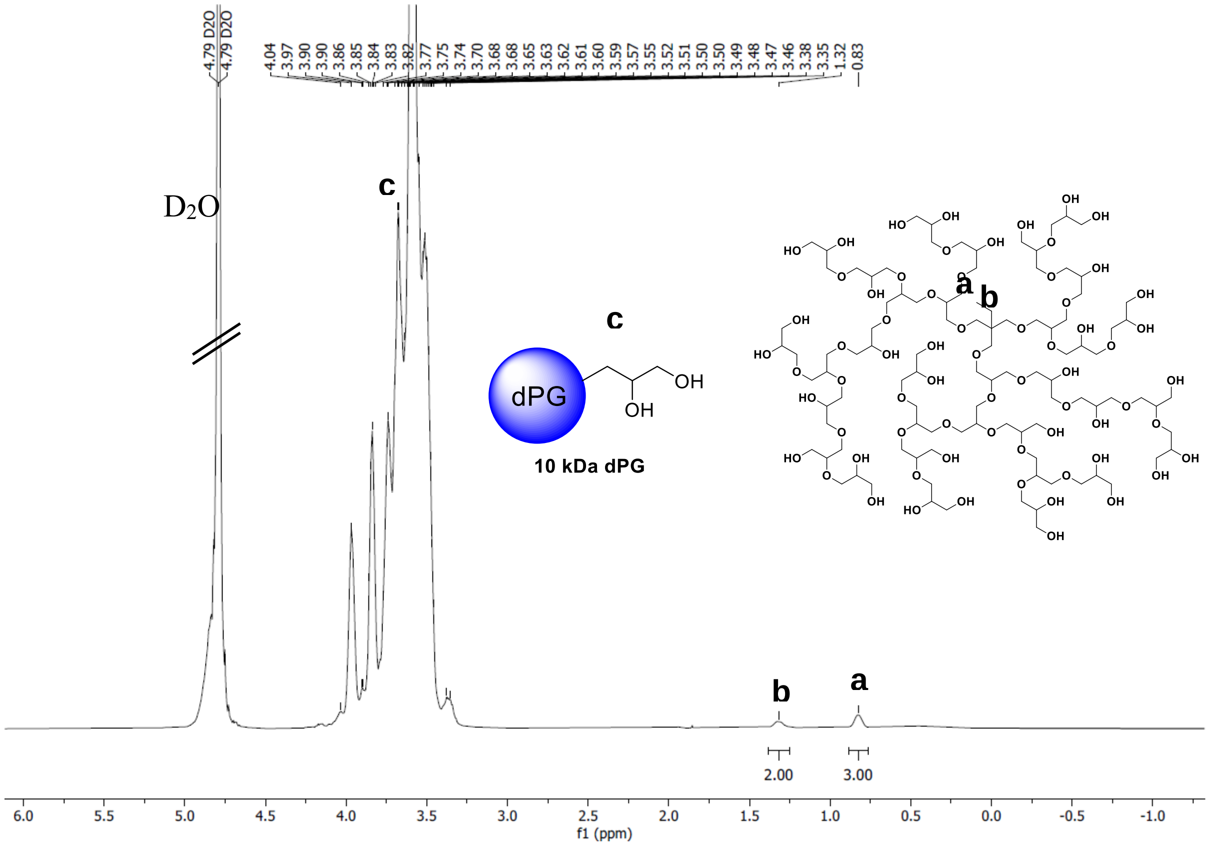

SI Figure 1 ^{1}H NMR (500 MHz, D_2O, δ (ppm)) of 10 kDa dPG (starting material)

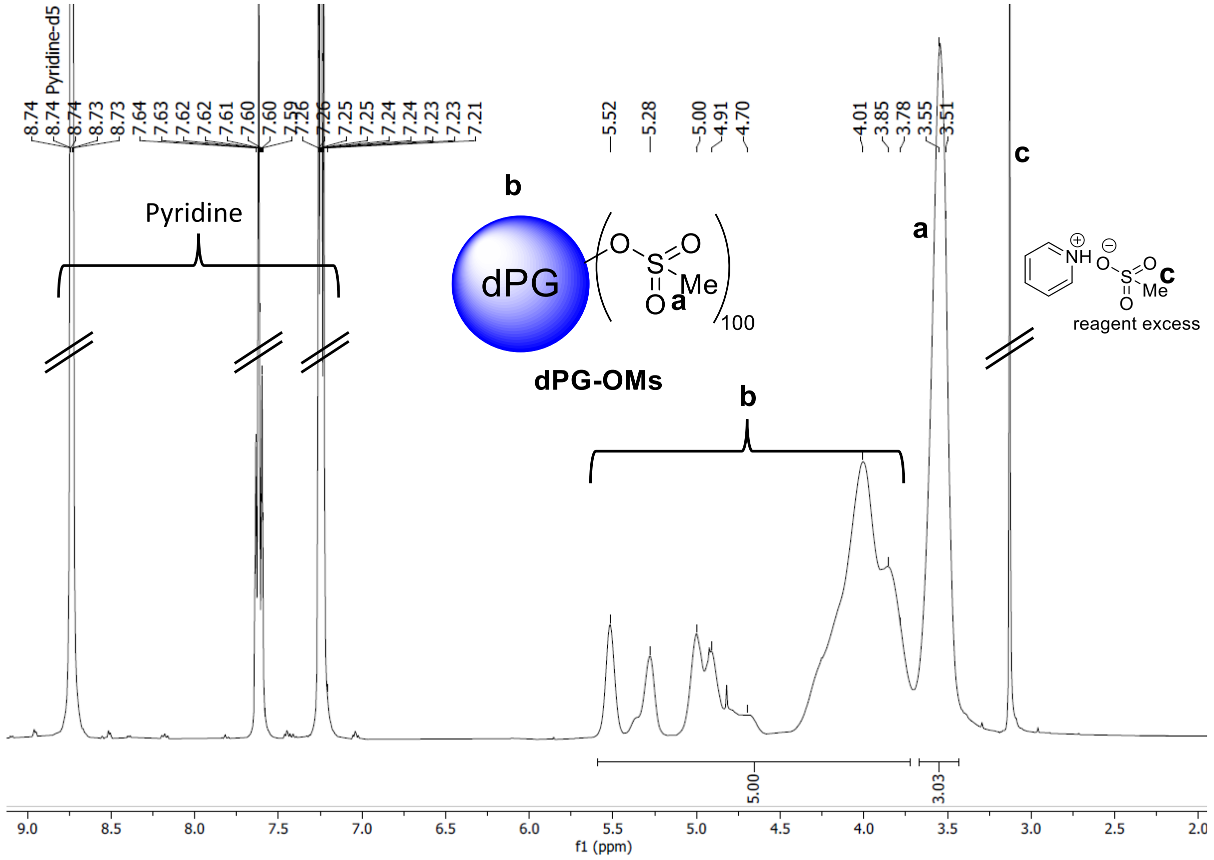

SI Figure 2: ^{1}H NMR (500 MHz, D_2O, δ (ppm)) of dPG-OMs

dPG-OMs was subsequently used in the next step without further purification. Sodium azide (405 eq., 202.5 mmol, 13.2 g) was then added to the *N,N*-Dimethyl formamide solution (50 mL) of dPG-OMs. The reaction suspension was heated to 60 °C and stirred for 3 days. Afterward, the crude mixture was cooled down by ice bath for 30 min and the precipitated was filtered off. The remaining filtrate was then subjected to dialysis with 1 kDa cutoff dialysis tube in chloroform for 2 days. After the purification, the product solution was dried and collected. The resulting viscous yellowish liquid was obtained with 80% yield. ^{1}H NMR (500 MHz, $CDCl_3$, δ (ppm)): 0.88 (3H, backbone initiator), 3.34 – 3.76 (m, backbone repeating units) (SI Figure 3). IR spectrum of dPG-Azide, showing the azide stretching at 2089 cm^{-1} (SI Figure 4).

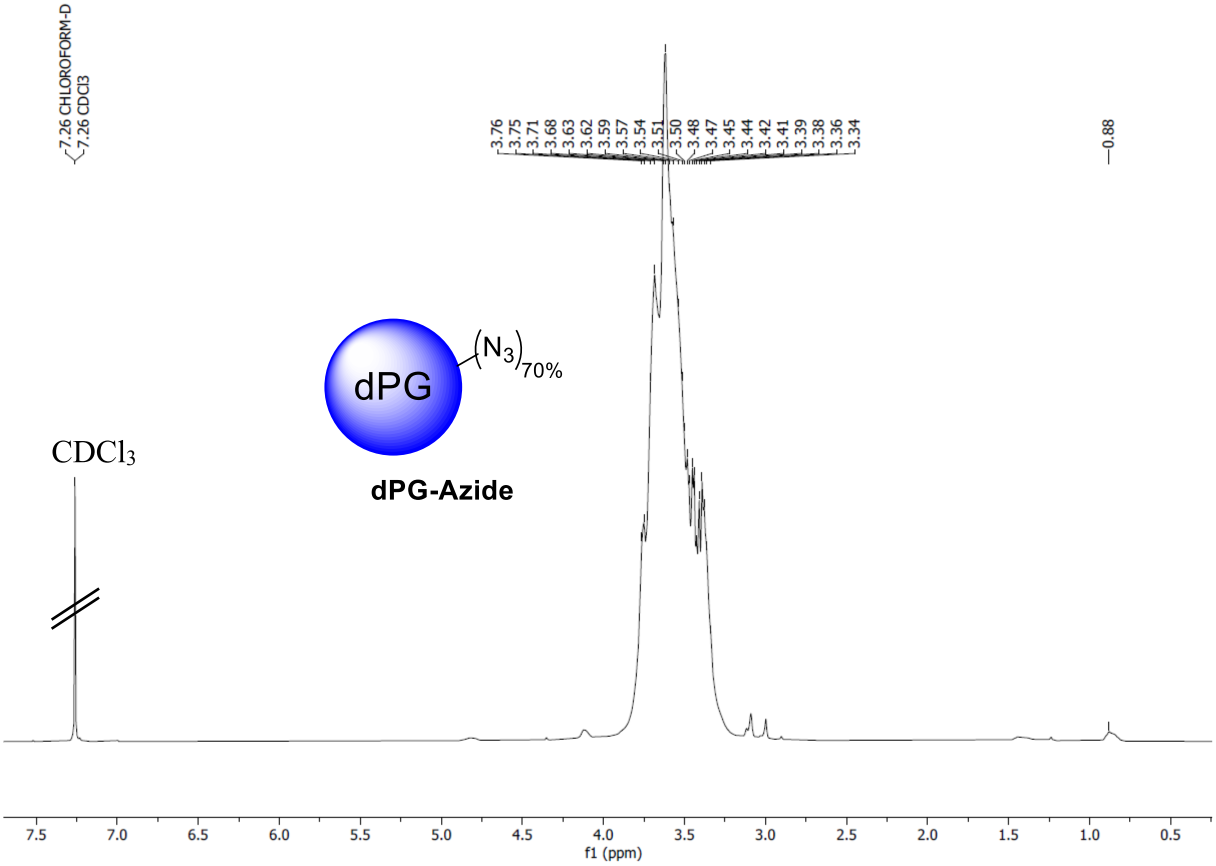

SI Figure 3: ^{1}H NMR (500 MHz, $CDCl_3$, δ (ppm)) of dPG-Azide

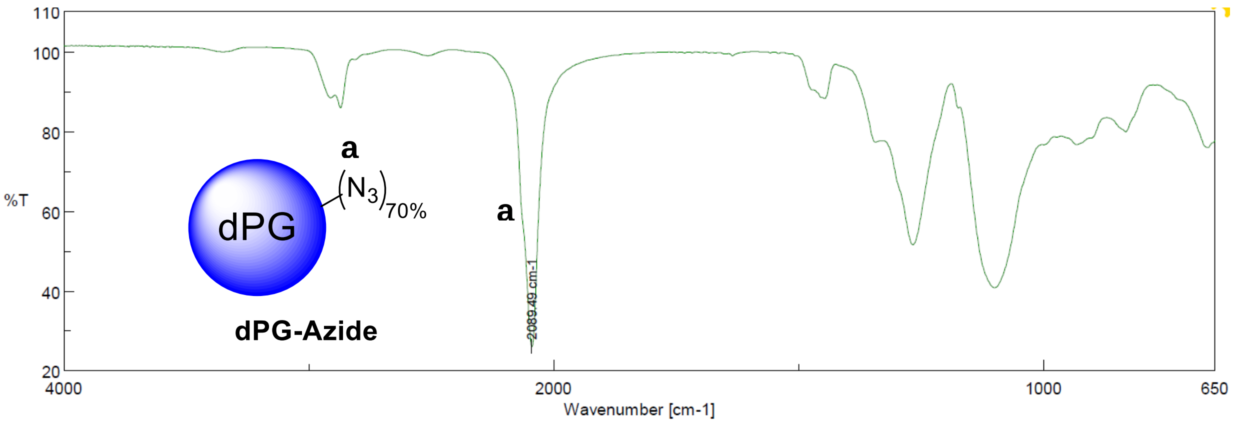

SI Figure 4: IR spectrum of dPG-Azide

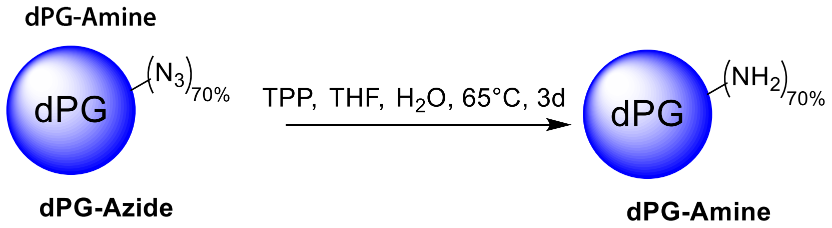

SI Scheme 2: Synthesis of dPG-Amine

triphenylphosphine (135 eq., 54 mmol, 14.2 g) was dissolved in 50 mL THF and added dropwise To the tetrahydrofuran (THF, 50 mL) solution of dPG-Azide (1 eq., 0.4 mmol, 4 g). Water was then added to the reaction mixture as long as the solution remains clear. The reaction was heated up to 65 °C and run for 3 day. Afterward, All solvents were removed from the crude mixture which was subsequently redissolved back with water. The aqueous solution was cooled down by ice bath for 30 min, making triphenylphosphine oxide byproducts precipitate which was later filtered off. The remaining solution was later purified by dialysis with 1 kDa cutoff in methanol for 1 day and water for 1 day respectively. The dPG-Amine product (yellowish aqueous solution) was then concentrated, collected and stored in water. After measuring the concentration of the stored product solution, its yield resulted in 85%. The percentage of amine groups was calculated as follows: ((**c**/2)+(**b**/1))/((**a**+**b**+**c**)/5)*100%. ^{1}H NMR (500 MHz, mixture of D_2O and DMF-d6, δ (ppm)): 2.86 – 3.19 (m, -CH_2-NH_2), 3.51 – 3.64 (m, CH-NH_2) 3.40 – 3.66 (m, backbone repeating units) (SI Figure 5). ^{13}C NMR (500 MHz, mixture of D_2O and DMF-d6, δ (ppm)): 36.96 – 43.50 (-CH_2-NH_2), 49.54 – 79.43 (backbone repeating units) (SI Figure 6)**.** IR spectrum showed no sign of azide peak anymore (SI Figure 7).

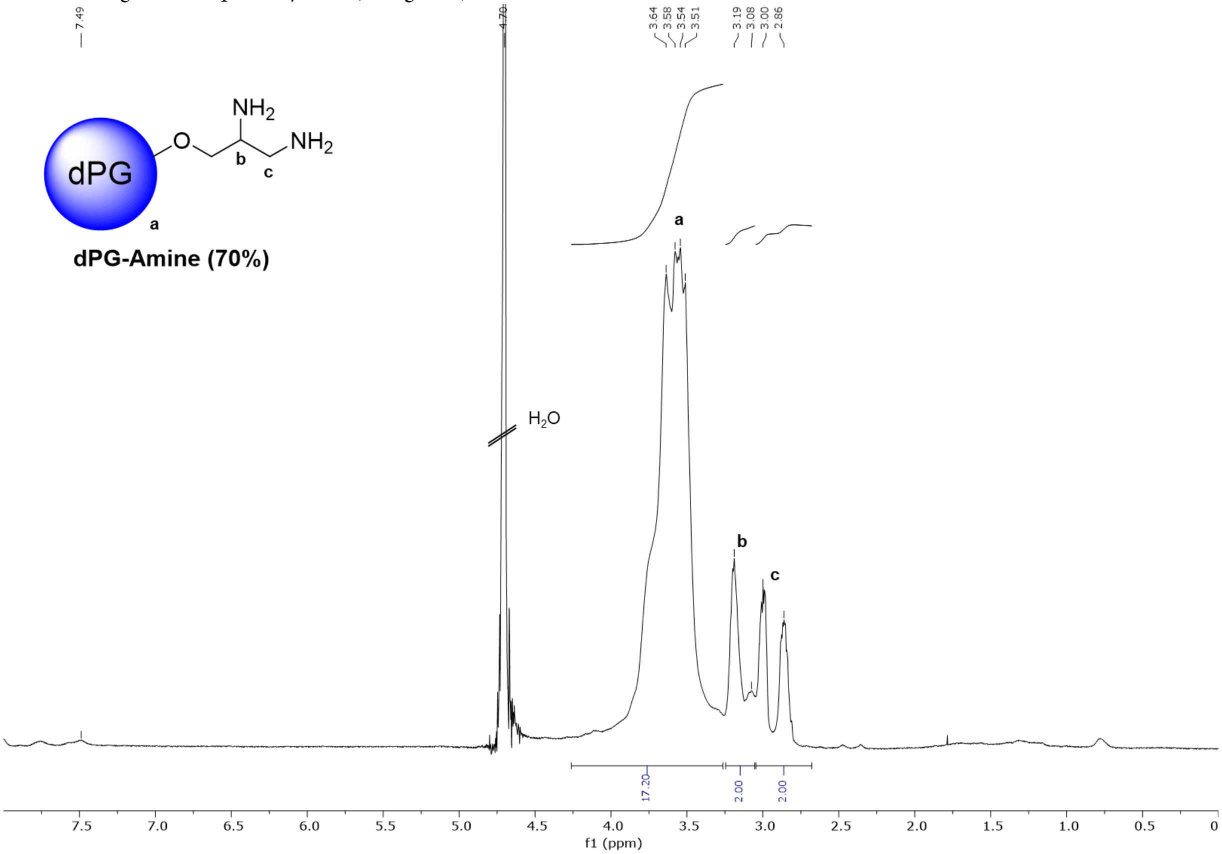

SI Figure 5: ^{1}H NMR (500 MHz, D_2O, δ (ppm)) of dPG-Amine

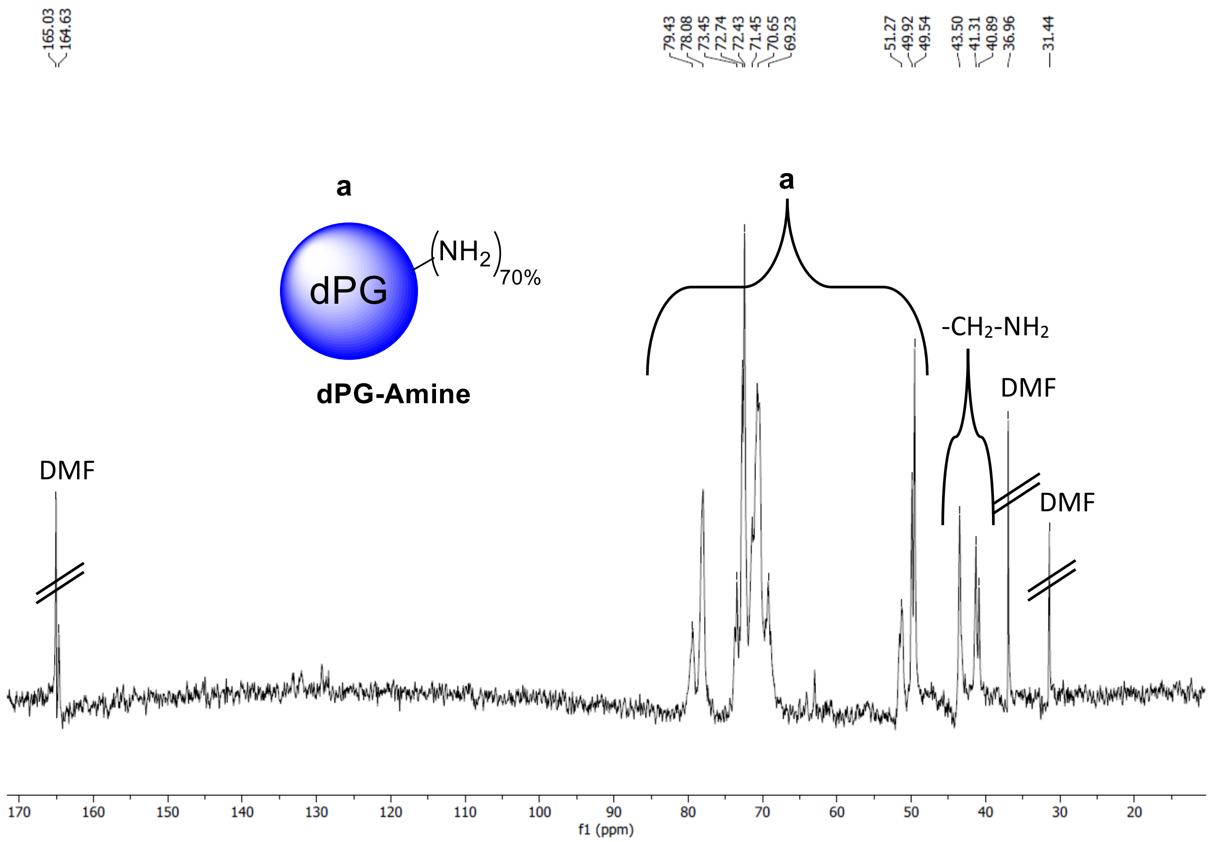

Figure 6: ^{13}C NMR (500 MHz, D_2O + DMF-d6, δ (ppm)) of dPG-Amine

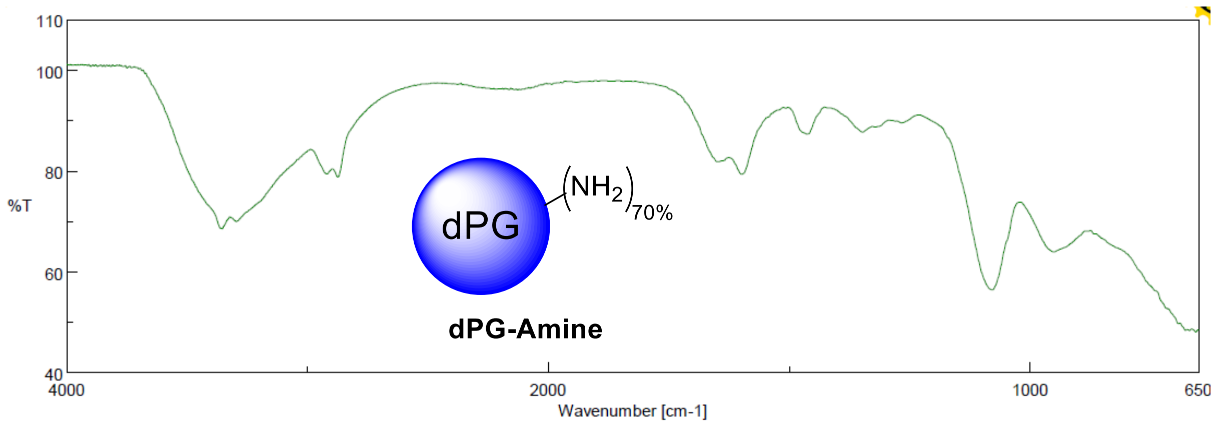

Figure 7: IR spectrum of dPG-Amine

dPG-C-mal

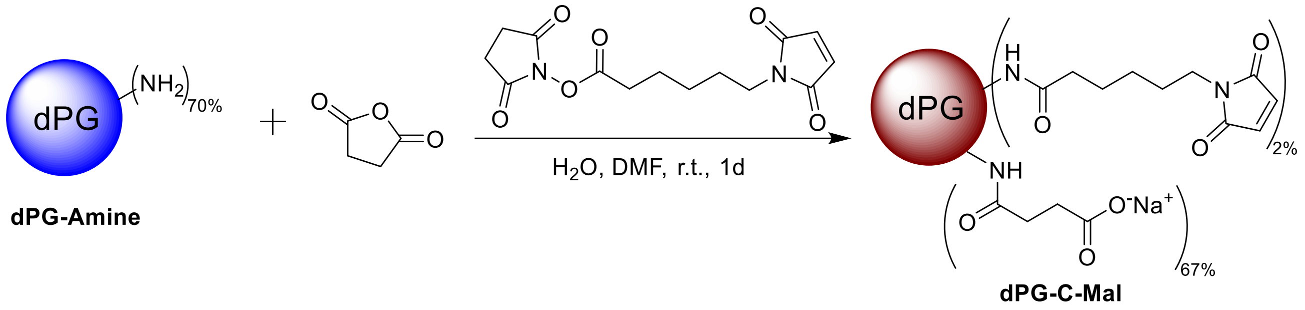

SI Scheme 3 Reaction scheme of dPG-C-mal synthesis.

Dendritic polyglycerol amine (dPG-Amine, 1 eq., 0.05 mmol, 0.5 g) was dissolved with 20 mL water in a 100-mL round bottle flask at room temperature. The acetone solution (10 mL) of succinic anhydride (110 eq., 5.5 mmol, 0.55 g) was then added dropwise into the reaction flask and the reaction was stirred for 1 h. Later, the pH of the reaction solution was adjusted to approximately 6-7 and the flask was cooled down to 0°C by using an ice bath. An excess of *N*-Succinimidyl 6-maleimidocaproate (10 eq., 0.5 mmol, 0.15 g) which was dissolved in DMF (5 mL) was added dropwise to the reaction flask and it was run for 1 day (SI Scheme 3). The crude mixture was the subjected to dialysis with 2 kDa cut-off in water for 2 days. Afterwards, the aqueous solution of the product was lyophilized to finally result in a yellowish lyophilized solid with a 71% yield. ^{1}H NMR (600 MHz, D_2O, δ (ppm)): 0.89 (3H, broad s, initiator backbone), 1.27 (2H, broad s), 1.58 (4H, broad s), 2.24 (2H, broad s), 2.48 – 2.83 (4H, broad s), 3.24 – 4.16 (m, backbone repeating units), 6.85 (2H, broad s) (SI Figure 8).

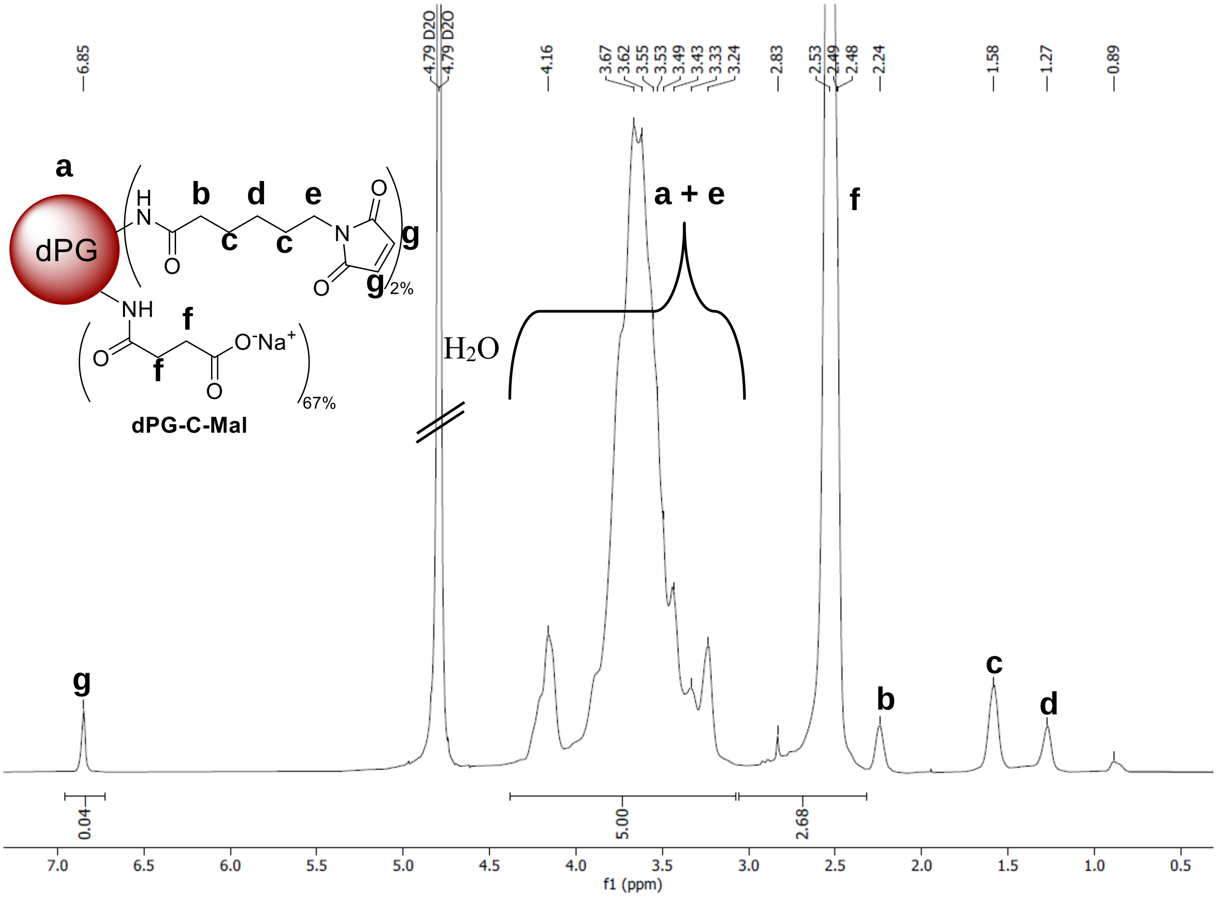

SI Figure 8: ^{1}H NMR (600 MHz, D_2O, δ (ppm)) of dPG-C-mal

PEG dithiol

$PEG(OMs)_2$

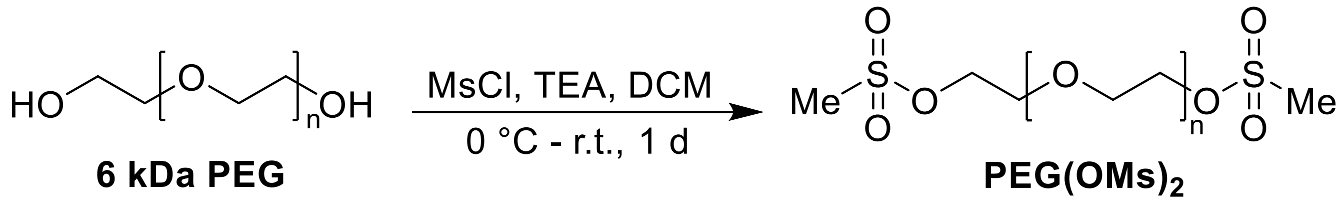

SI Scheme 4: Synthesis of $PEG(OMs)_2$

6 kDa polyethylene glycol (PEG, 1 eq., 16.7 mmol, 100 g) was first dried under vacuum at 70 °C overnight. Later the dried PEG was dissolved in dichloromethane (DCM, 500 mL) and was cooled down by ice bath. Triethylamine (TEA, 5 eq., 83.3 mmol, 11.6 mL) was added to the reaction flask, followed by the dropwise addition of MsCl (3.5 eq., 58.3 mmol, 4.5 mL). The reaction was run for 1 day. The crude mixture was later washed with brine trice and dried with Na_2SO_4, followed by precipitation by cooled ether. The white precipitate yielded 95%. ^1H NMR (500 MHz, $CDCl_3$, δ (ppm)): 3.08 (3H, s), 3.48 - 3.78 (m), 4.37-4.38 (2H, t) (**SI Figure 9**).

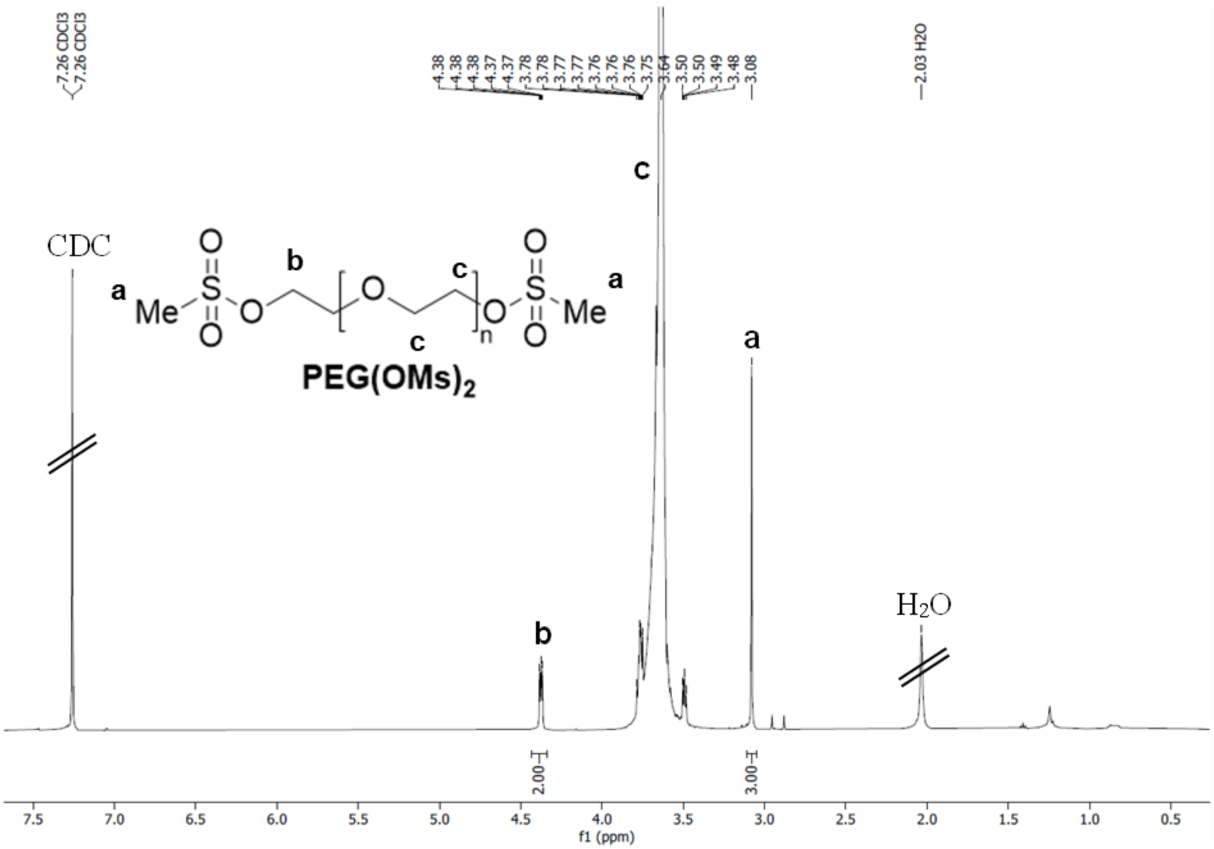

SI Figure 9: ^1H NMR (500 MHz, $CDCl_3$, δ (ppm)) of PEG(OMs)

PEG dithiol

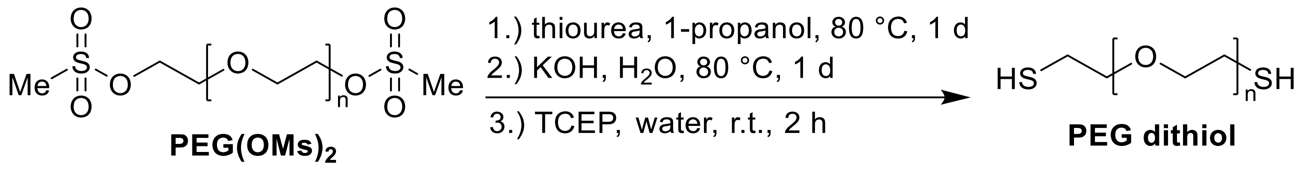

SI Scheme 5: Synthesis of PEG dithiol

$PEG(OMs)_2$ (1 eq., 15.8 mmol, 95 g) was dissolved in 1-propanol (500 mL). Thiourea (8 eq., 126.7 mmol, 9.6 g) was added into the reaction flask which was then heated to 80 °C and stirred for 1 day. The next day, 1-propanol was removed and the crude mixture was redissolved back with deionized water (500 mL). KOH was then added and the flask was heated up to 80 °C and stirred for 1 day. The Tris(2-carboxyethyl)phosphine hydrochloride (TCEP, 2 eq., 31.7 mmol, 9.1 g) was then added to the solution and it was run for 2 h before purification. The final crude product was first extracted by DCM trice, dried with Na_2SO_4 and concentrated by rotary evaporator. The extract was then precipitated in cooled ether to finally result in the pale yellowish precipitate with 84% yield. ^1H NMR (500 MHz, $CDCl_3$, δ (ppm)): 1.57 – 1.61 (1H, t), 2.67 – 2.71 (2H, quat), 3.48 - 3.78 (m). (Figure S10). The number of thiol group was quantified by Ellman assay from *Thermo Fischer Scientific* using a cysteine calibration curve. The result showed that there are approximately 1.95 groups of thiol per chain.

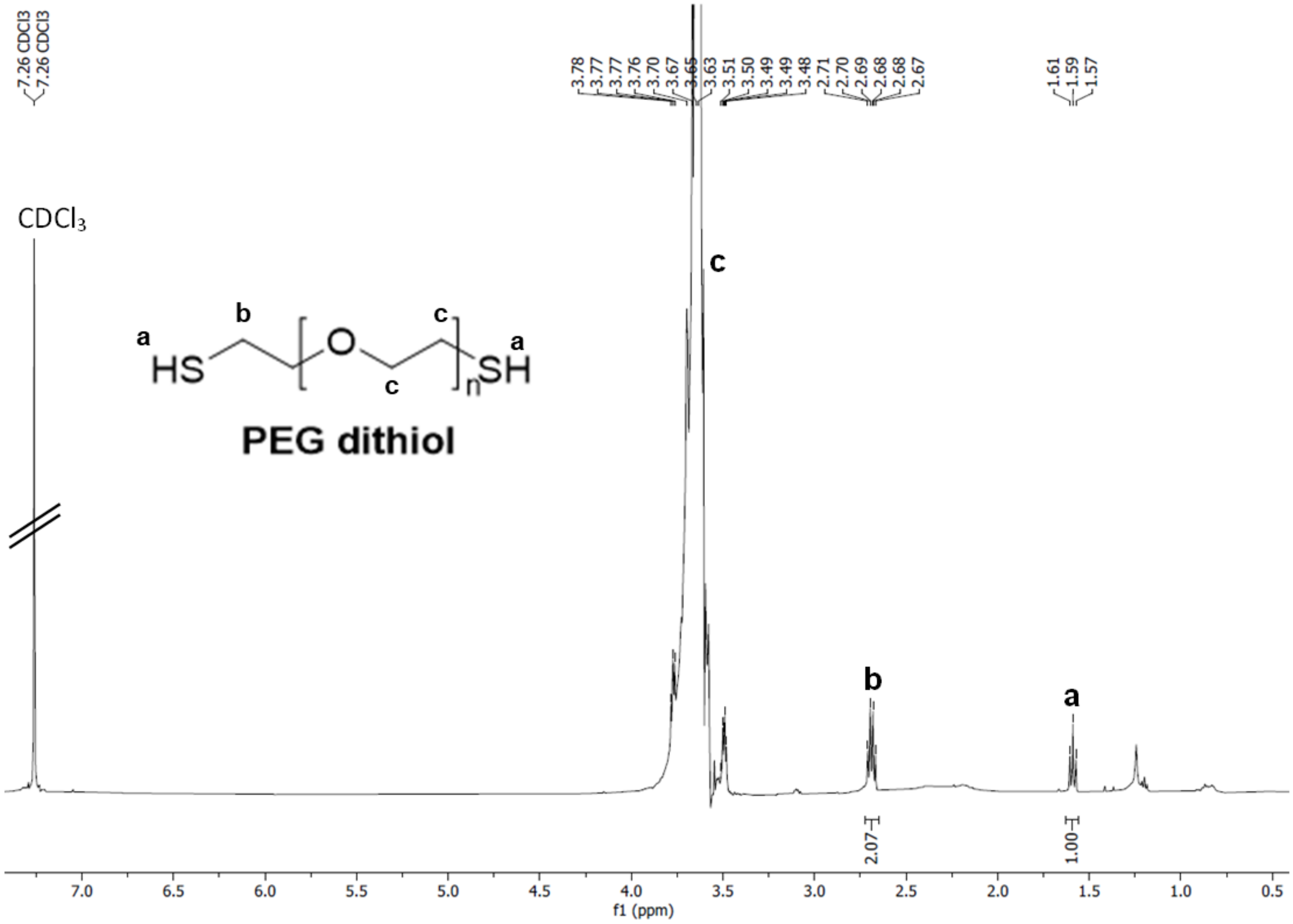

SI Figure 10: ^1H NMR (500 MHz, $CDCl_3$, δ (ppm)) of PEG dithiol

NMR of formed gel

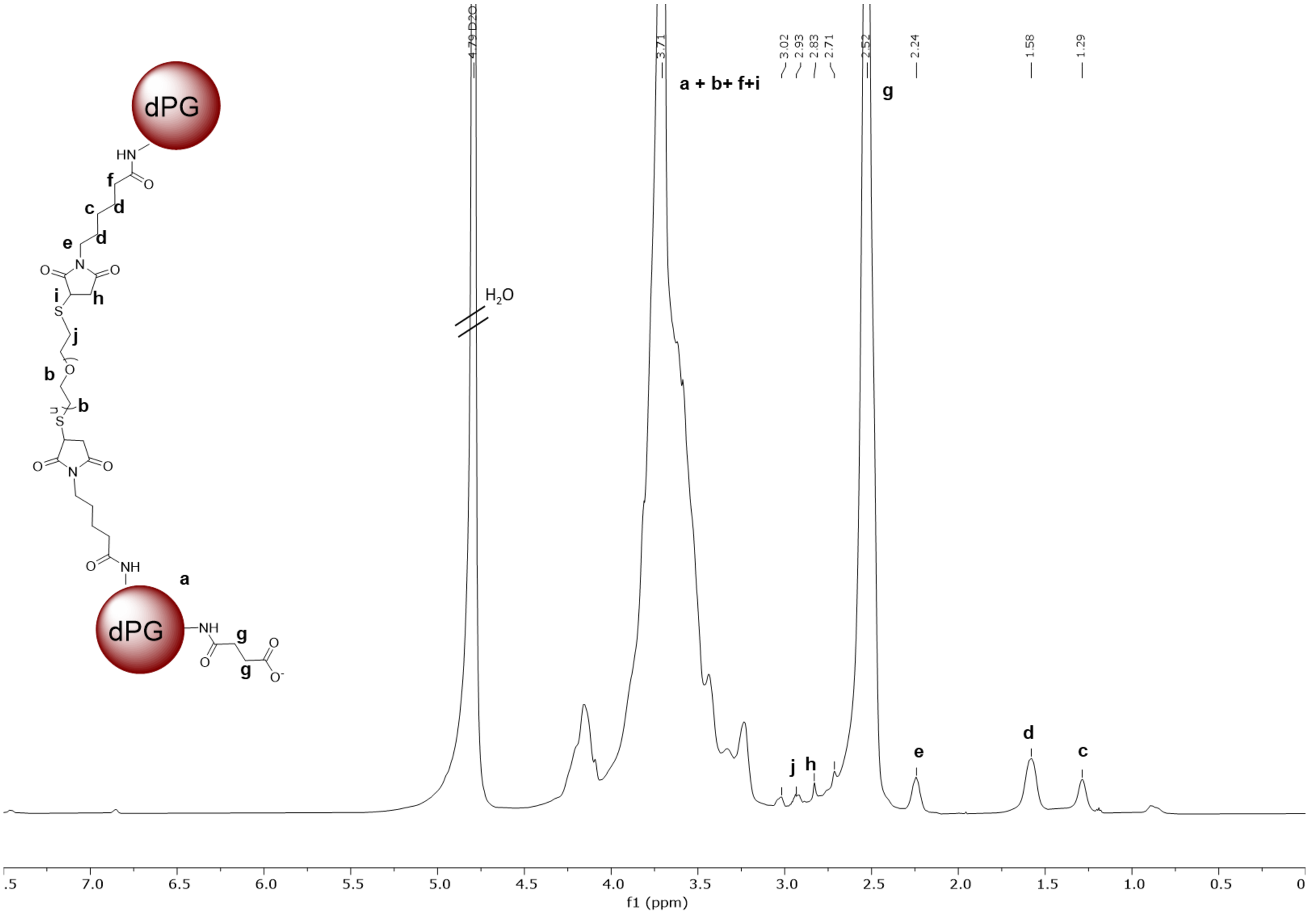

SI Figure 11: ^{1}H NMR (700 MHz, D_2O, δ (ppm)) of formed gel

The gel was formed as described in Table 1 using D_2O as a solvent. ^{1}H NMR (600 MHz, D_2O, δ (ppm)): 0.89 (3H, broad s, initiator backbone), 1.29 (2H, broad s), 1.58 (4H, broad s), 2.24 (2H, broad s), 2.48 – 2.83 (4H, broad s), 3.24 – 4.16 (m, backbone repeating units of PG and PEG).

Surface activation, Ellipsometry and AFM measurements

SI Table 1: Gelation

	V (PEG-dithiol) [µL]	V (dPGC-mal) [µL]	V (PBS) [µL]	c (Gel) [% w/v]	Gelation	t [min]
**Gel 1**	10	12	78	2.2	No	60
**Gel 2**	10	30	60	4	Liquid	50
**Gel 3**	10	40	50	5	Liquid	50
**Gel 4**	10	50	40	6	Vsicous	30
**Gel 5**	20	50	30	7	Viscous	10
**Gel 6**	30	60	10	9	Solid	10
**Gel 7**	30	70	0	10	solid	10

The components were mixed after dissolving the dPGC-mal or PEG-dithiol in PBS pH 8 to 10 w % solutions. Gel 5 yielded a suitable viscosity and gelation time for the spincoating process.

Goniometer measurements

Water contact angle measurements were performed on a goniometer OCA20 (Dataphysics Instruments, Filderstadt, Germany) to determine the best surface activation strategy, assuming a low contact angle induces a better hydrogel spread on the surface. The lowest contact angle was reached by Piranha solution activation. Thus, this method was chosen for surface activation.

SI Table 2: Surface activation

	Untreated gold	UV radiation	Piranha solution
**Contact angle** [°]	98 ± 1	75 ± 6	58 ± 13

Surface activation of the SPR biosensors

The SPR Chips were activated with freshly prepared piranha solution (75 % H_2SO_4, 25 % H_2O_2) for 30 mins and subsequently washed with deionized water.

Coating of the SPR biosensors

10 wt% solutions of respectively PEG-dithiol and dPGC-mal were prepared in PBS (pH = 8) and then mixed as described in table 1. The mixture was vortexed for 30 s and subsequently pipetted onto a gold coated glass slide (SIA kit AU, GE). Then, spincoating on a SPIN150-v3 spin coater was performed at 2 speed settings: first 180 s at 600 RPM and then 120 s at 1500 RPM. The Chips were then washed over night in deionized water.

Gel thickness determination by spectroscopic ellipsometry

The Ellipsometric dry and wet thicknesses were measured by a SENpro spectroscopic ellipsometer from SENTECH Instruments GmbH (Berlin, Germany) at an incident angle of 70° and wavelengths from 400 to 800 nm. Wet state measurements of the coated silicon wafers were performed in a buffer chamber using standard HBS-EP buffer. For each coated surface, three independent surfaces were used, and on each surface, three different spots were measured. Spectra were fitted to a Cauchy-layer fit. AFM was used to determine the wet state thickness of the coated SPR-Chip, as it was too thin to be fixed in the wet-state Ellipsometry Chamber.

Wet state gel thickness determination by Atomic force microscopy

Atomic force microscopy measurements were performed on a Multimode 8 atomic force microscope (Bruker, MA, USA) in tapping mode. The measurements were performed using SNL-10 cantilevers from Bruker with a spring constant of 0.35 N m^{-1} and a resonance frequency of 165 kHz. Scan frequency and amplitude setpoint were adjusted to achieve a maximum resolution. Obtained images were analyzed using the software NanoScope Analysis from Bruker (version 1.5) and processed using 1st order flattening and plane fit. The blank subtracted hydrogel thickness was determined by performing a cut on the surface using a scalpel and obtaining images around the cut. The height difference between the surrounding area and the lowest point of the cut was taken as the thickness of the hydrogel, given that the scalpel can cut through the whole hydrogel layer. This thickness was then subtracted from the thickness obtained after cutting the gold layer itself. Images were obtained in wet state by applying standard HBS-EP buffer in a wet measurement chamber.

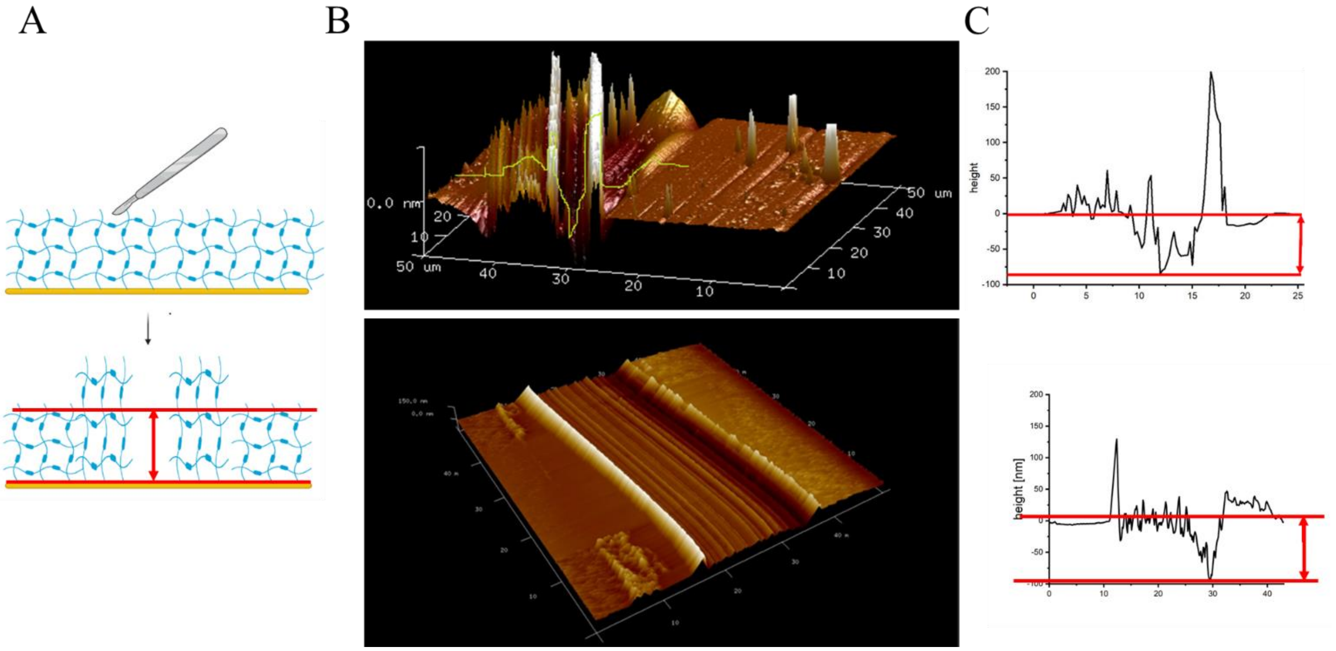

SI Figure 12: A) scheme of thickness determination by AFM. The coated chip was cut with a scalpel. Then, an AFM micrograph was performed in wet state. The thickness was defined as the distance from the lowest point to the even surface. Then, the thickness of the blank cut was subtracted to determine the hydrogel layer thickness. B) AFM micrographs of two cuts into dPG-PEG biosensors. C) Surface Profile of the dPG-PEG biosensors.

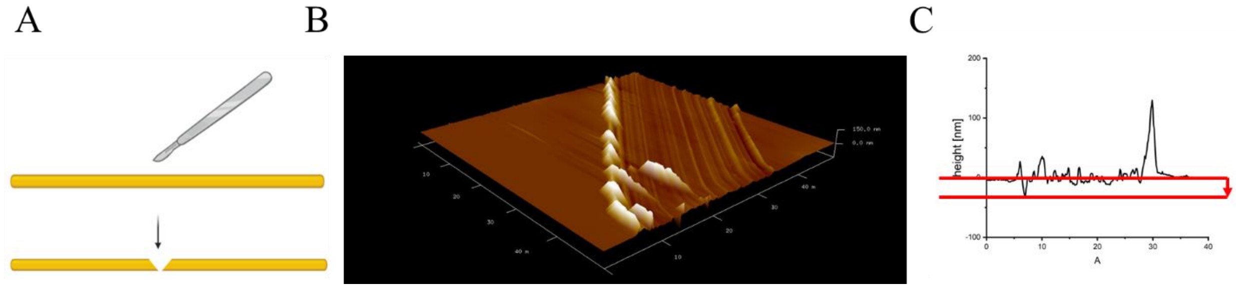

SI Figure 13: A) Scheme of blank cut into the gold layer for the blank thickness determination. B) AFM micrograph of the blank cut into gold sensor. C) Surface profile of the blank cut into gold sensor

Surface morphology analysis

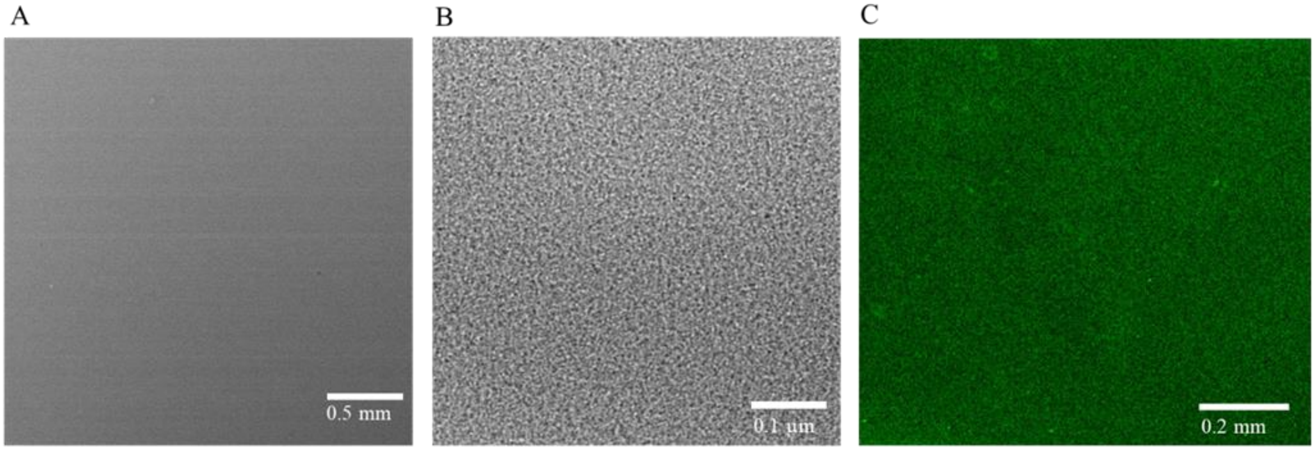

SI Figure 14: Gel morphology and mesh size determination. A) Scanning electron microscopy (SEM) image showing a homogenous gel distribution over the surface. B) Cryogenic transmission electron microscopy (Cryo-TEM) image of a hydrogel-coated gold grid. C) CLSM image of a Fluorescein isothiocyanate (FITC)-labeled antibody immobilized on a dPG-PEG hydrogel-coated chip proving a homogeneous distribution of immobilized antibody.

Cryo-TEM imaging

Perforated gold film-covered microscopical 300 mesh gold grids (UltrAuFoilR1.2/1.3 batch of Quantifoil, MicroTools GmbH, Jena, Germany) were hydrophilized by 60 s glow discharging at 10 mA in a Safematic CCU-010 device (safematic GmbH, Zizers, Switzerland).Subsequently, the grids were spin-coated and washed as described above, with a reduced gel volume of to 2 µL to retain the same gel volume to surface are ratio. After drying, 4 µL buffer solutions were applied to the grid to hydrate the hydrogel film. The samples were vitrified by automatic blotting and plunge freezing with a FEI Vitrobot Mark IV (Thermo Fisher Scientific Inc., Waltham, Massachusetts, USA) using liquid ethane as cryogen. The vitrified specimens were transferred to the autoloader of a FEI TALOS ARCTICA electron microscope (Thermo Fisher Scientific Inc., Waltham, Massachusetts, USA). This microscope is equipped with a high-brightness field-emission gun (XFEG) operated at an acceleration voltage of 200 kV. Micrographs were acquired on a FEI Falcon 3 direct electron detector (Thermo Fisher Scientific Inc., Waltham, Massachusetts, USA) using a 100 µm objective aperture.

Partition coefficient determination by diffusion analysis of FITC-labeled Dextran

The partition coefficient of dextrans into the dPG-PEG-based hydrogel was determined as previously described.[5] In brief, the hydrogel was placed on a glass coverslip and incubated with HBS-EP solution containing FITC-labeled dextran. After an incubation time of 30 min, confocal microscopy was used to determine the dextran concentration in the middle of the hydrogel perpendicular to the glass interface (z-axis). These measurements were performed with dextrans with molecular weights ranging between 4 kDa and 70 kDa (Panels A-E), corresponding to Stokes radii of ~ 1.4 to 6.0 nm, respectively. Each panel contains 10 concentration traces (drawn using different colors), which have been recorded with a delay time of 15 s. In all measurements, these curves superimpose very well, indicating that the dextran distribution is in equilibrium in both phases (i.e., the incubation time of 30 min is sufficient to reach equilibrium, which was expected from previous measurements on a related hydrogel [5]. The partition coefficient is given by the dextran concentration value within the hydrogel (c) normalized by the bulk concentration (c bulk) and decreases monotonously with increasing dextran size.

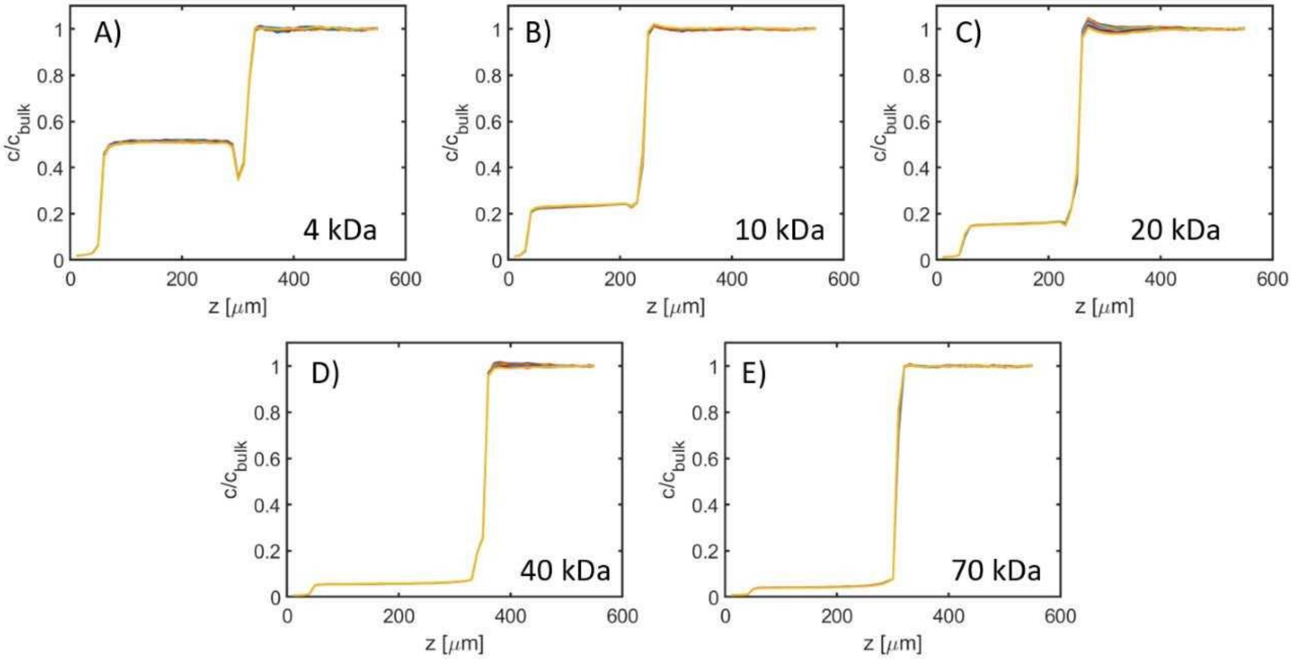

SI Figure 15: Concentration traces between the hydrogel and solution of dextrans of different sizes (A-E) after 30 mins of incubation time.

SPR Measurements for biosensor validation

All experiments were performed on a BiaCoreX100 (GE Healthcare) at 25°C. The Chips were preconditioned with 30 s pulses of 1 M NaCl 50 mM NaOH solution at 30 µL/min.

Preconcentration scouting:

To determine optimal loading conditions of the CMD-Chip, CAII (Merck) (50 µg/mL) were injected on the CMD Biosensor (CM5 – GE Healthcare) at pH 4.0; pH 4.5; pH 5.0; pH 5.5. The steepest slopes were reached for pH 4.5 or pH 5.0, however resulting binding sensorgrams of CAII immobilized at pH 4.5 (B) or pH 5.0 (C) showed reduced binding signal due to denaturation of CAII. Thus, pH 5.5 was chosen as the immobilization buffer and yielded the full expected acetazolamide binding response (Figure 3C.1).

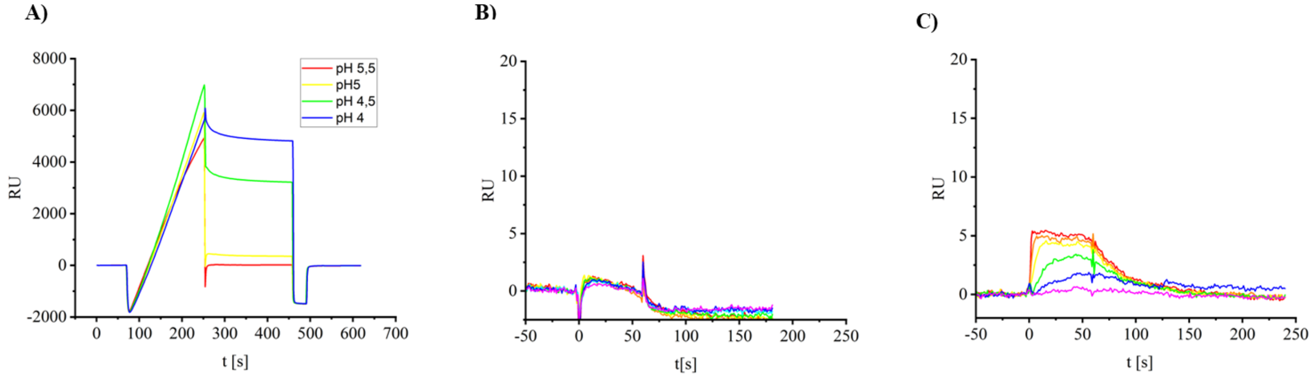

SI Figure 16: A) Sensorgrams of preconcentration scouting on CMD biosensor B) Acetazolamide binding to CAII immobilized at pH 4.5 C) Acetazolamide binding experiment to the CAII immobilized at pH 5.0.

Immobilization

Carbonic anhydrase II (CA II) was diluted to a 50 µg/mL solution in 10 mM sodium acetate buffer pH 5.5 and injected onto EDC/NHS (GE Healthcare) activated preconditioned CMD biosensors or PG-PEG biosensors. The remaining active esters were quenched with a 1 M Ethanolamine solution (GE Healthcare). 130 µL (maximum injection Volume) of CA II-solution were injected at a flow rate of 5 µL/min. To compare the binding performance, 3400 RU of CAII (maximum loading on a CM5 Chip) were also immobilized on a PG-PEG biosensor by injecting pulses of CAII-solution using the Biacore™ X100 control software.

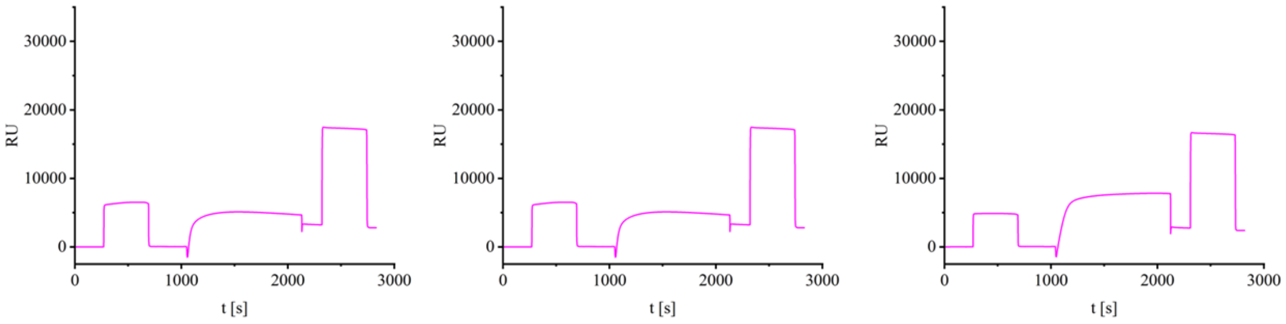

SI Figure 17: Sensorgrams of maximum CAII immobilization on three CMD biosensors.

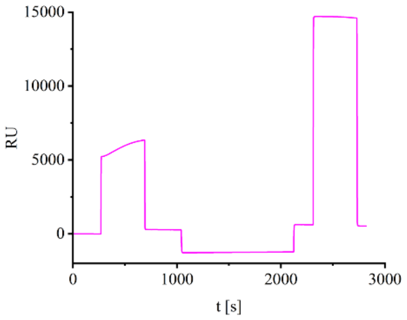

SI Figure 18: Sensorgram of maximum CAII immobilization on a PEG biosensor (cytiva). 345 RU were immobilized.

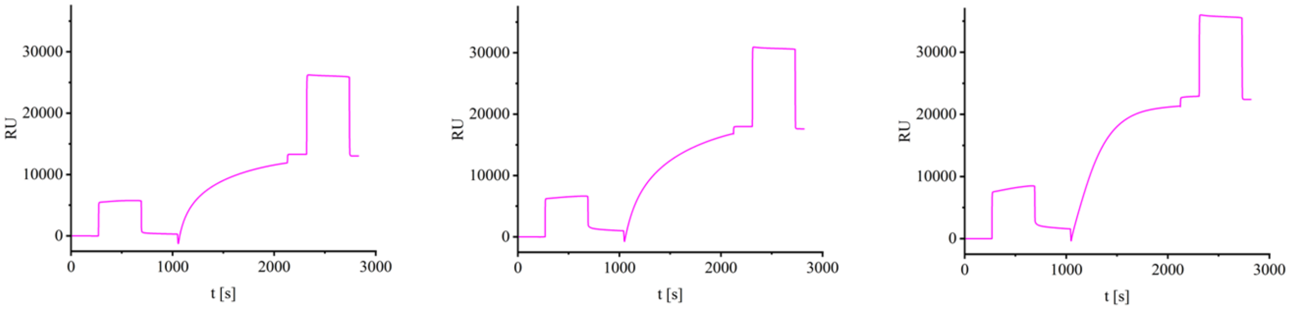

SI Figure 19: Sensorgrams of maximum CAII immobilization on three PG-PEG biosensors.

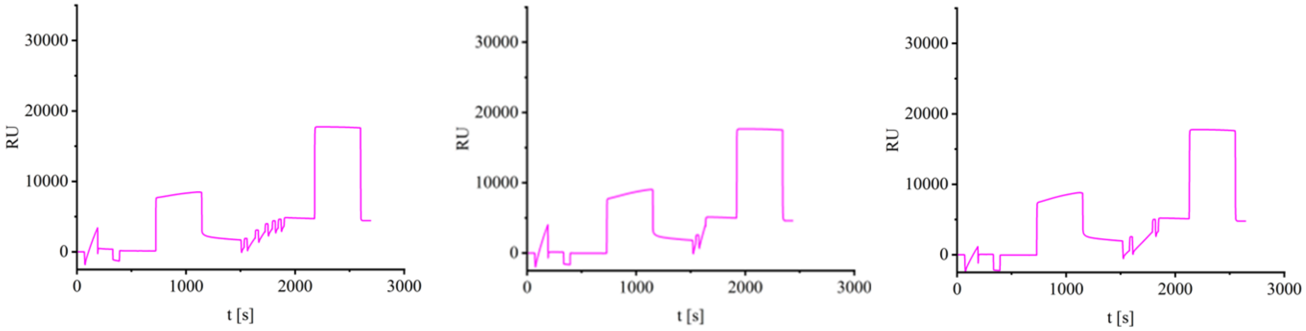

SI Figure 20: Sensorgrams of pulse-mediated immobilization on a PG-PEG biosensor until 3400 RU of CA II were immobilized. Here, 3400 RU was entered as a target for immobilized protein in the Biacore X100 control software

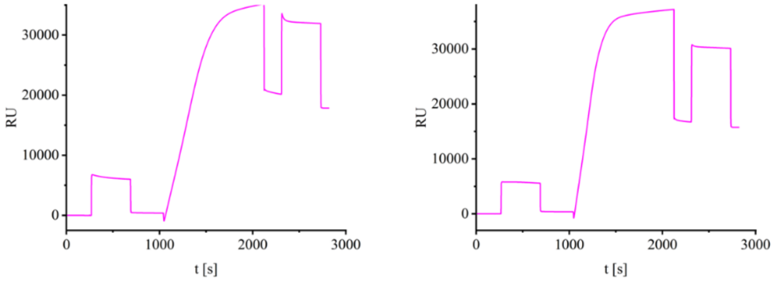

SI Figure 21: Sensorgrams of maximum loading of CM7 biosensors with CA II (n =2). Here, an average of 16419 ± *1.0* RU was reached (n =2).

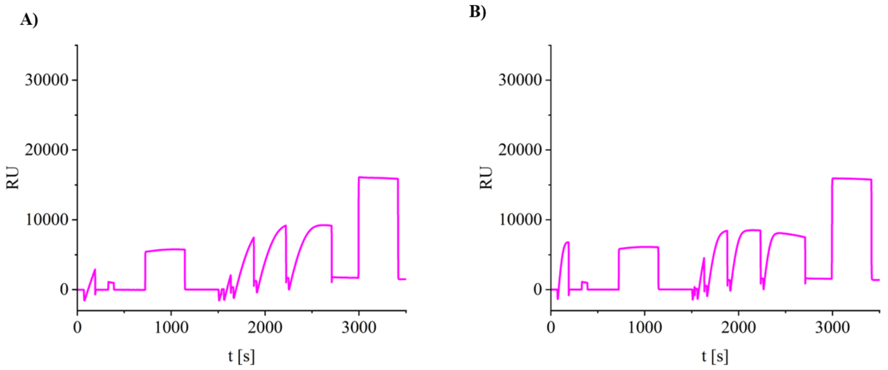

SI Figure 22: Sensorgrams of pulse mediated immobilization of 25 μg/mL (A) and 50 μg/mL (B) of CA II on a CMD biosensor using the Biacore X100 control software to determine the amount of necessary protein for 3400 RU of CA II loading. Here, 3400 RU was entered as a target for immobilized protein in the software. However, only 1362.1 RU were reached in (A) and 1473.2 RU were reached in (B) after all of the 130 μL were injected. This suggests that the immobilization process works much better by using the continuous injection mode, possibly because of the longer presence of a high CA II concentration in the CMD matrix.

Acetazolamide (Thermo Fisher Scientific) was dissolved in 50 mM NaOH-solution and then diluted in Hepes buffer saline containing EDTA and 0.005 % Polysorbat (HBS-EP) (GE Healthcare) to a 1000 nM solution. Triplicate concentration series of 5 different concentrations were created by 1:2 dilution and injected at an association time of 60 s and a dissociation time of 180 s at a flow rate of 10 μL/min. longer dissociation times were chosen for the maximum loaded PG-biosensors to enable a full dissociation of analyte depending on the loading level. Binding constants were determined by fitting to a 1:1 kinetic model using the BIAevaluation software (GE Healthcare).

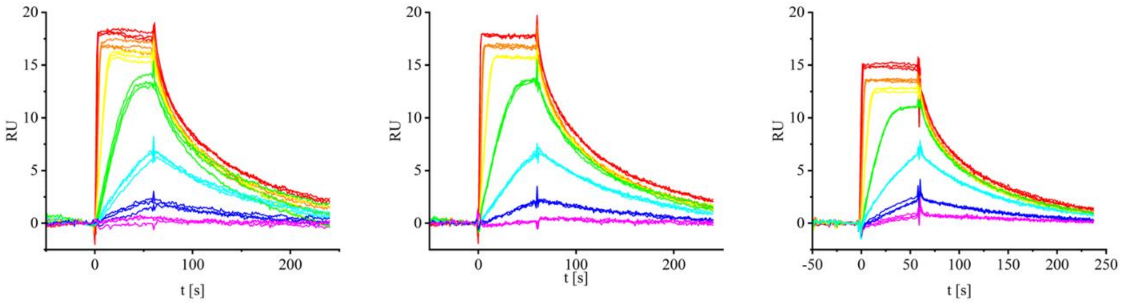

SI Figure 23: Sensorgrams of binding experiments on three max loaded CMD biosensors

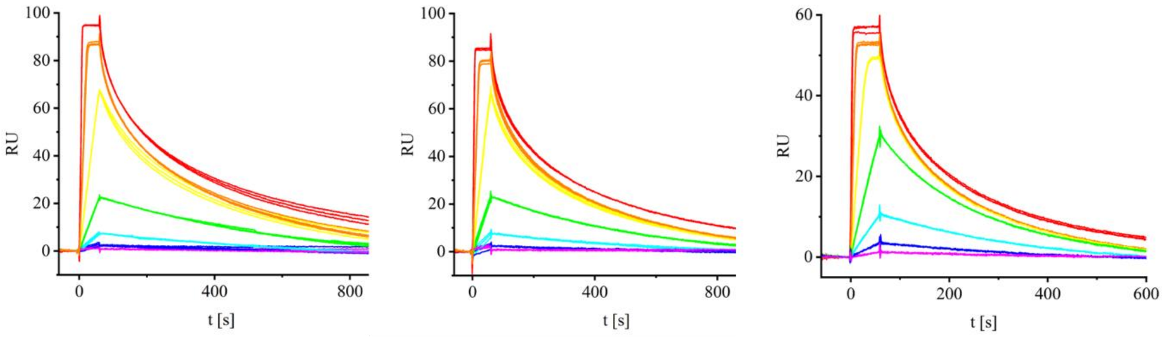

SI Figure 24: Sensorgrams of binding experiments on three max loaded PG-PEG biosensors

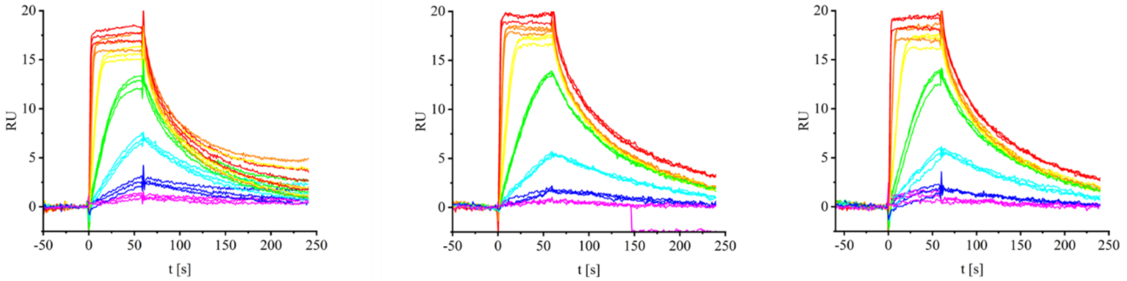

SI Figure 25: Sensorgrams of binding experiments on three 3400 RU CAII loaded PG-PEG biosensors

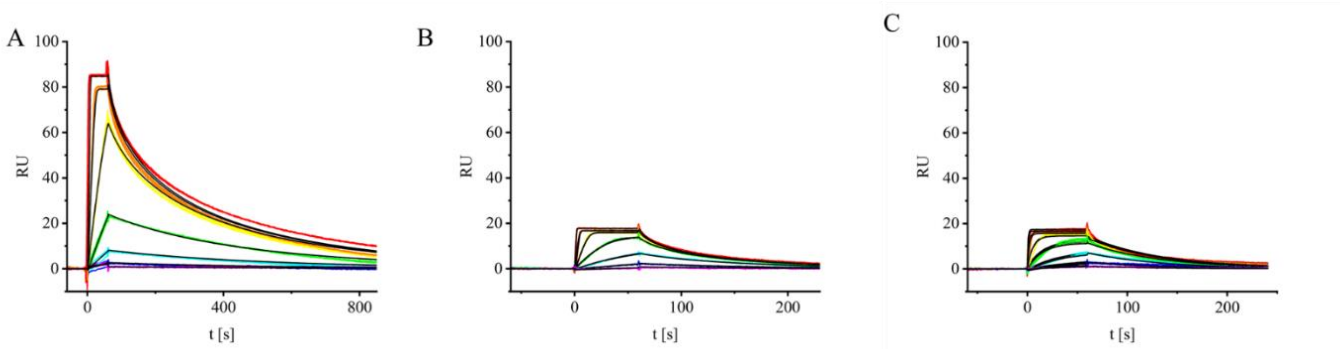

SI Figure 26: Representable examples of 1:1 kinetic fits for K_D determination. Sensorgrams show acetazolamide binding to: A: maximum immobilized CAII on PG-PEG chip B) maximum immobilization on CMD Chip C) 3000 Target RU immobilized on PG-PEG Chip. Black lines are the fitted curves.

SPR Assays- IgG Interactions

Immobilization

Mouse IgG2b kappa (Invitrogen) was diluted to 25 μg/mL in 10 mM sodium acetate buffer (pH = 5) and injected onto EDC/NHS activated preconditioned biosensor. The remaining active esters were quenched with a 1 M ethanolamine solution. Mouse IgG2bκ was injected in pulses until 300 RU were reached.

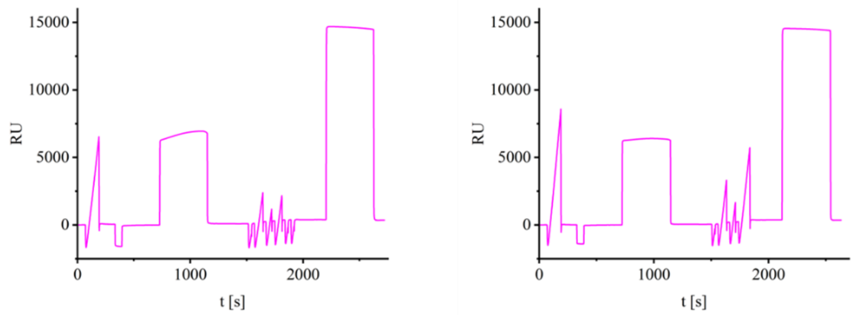

SI Figure 27: immobilization of 300 Target RU Mouse IgG2bκ on two CMD-biosensors.

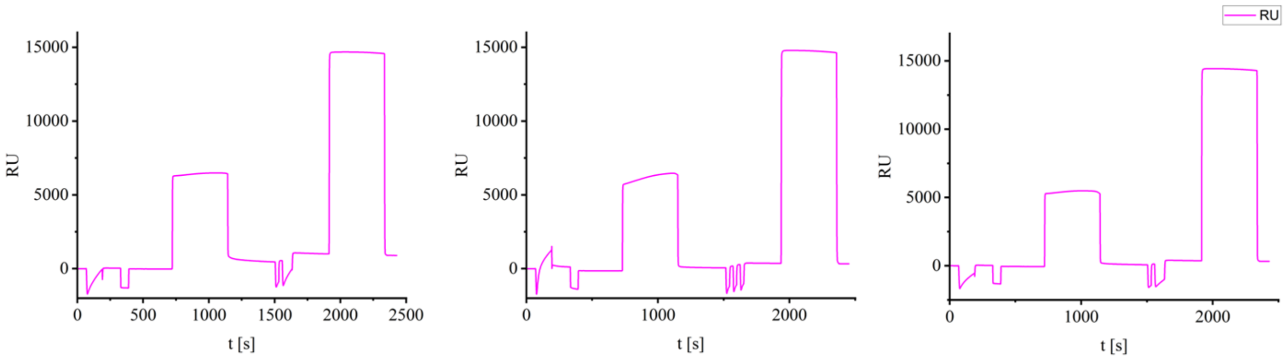

SI Figure 28: immobilization of 300 target RU Mouse IgG2bκ on 3 PG-PEG biosensors

FITC anti-mouse IgG2b (Biolegend clone RMG2b-1) was dialyzed against HBS-EP solution to yield a 5 µg/mL solution. Triplicate concentration series of five different concentrations were prepared by 1:1 dilution (5 µg/mL, 2.5 µg/mL, 1.25 µg/mL, 0.625 µg/mL, 0.3125 µg/mL) and injected at an association time of 150 s and a dissociation time of 600 s. A 30 s pulse of 10 mM Glycine-HCl solution (pH = 2) was used to regenerate the gel after each cycle.

Binding experiments on 300 RU immobilized Sensors

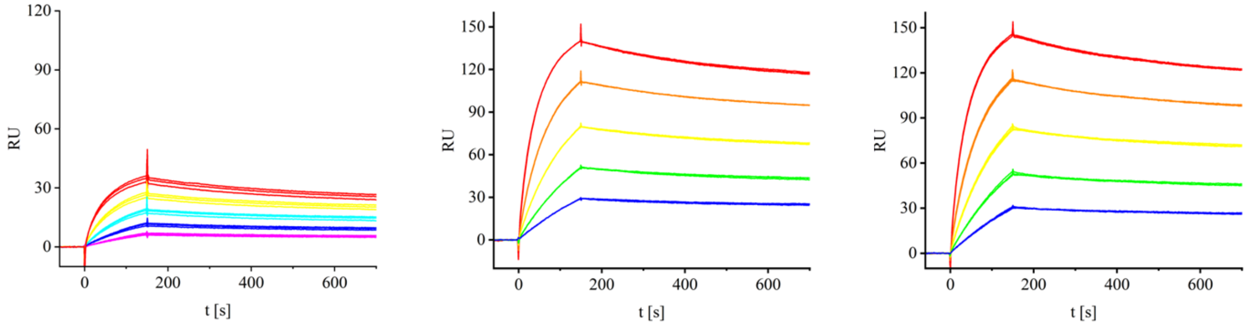

SI Figure 29: Sensorgram of the binding of FITC anti-mouse IgG2b to mouse IgG2b kappa on three CMD biosensors. The maximum response variation likely results from limited stability of mouse IgG2bkappa at the immobilization pH. However, this does not influence the resulting binding constant.

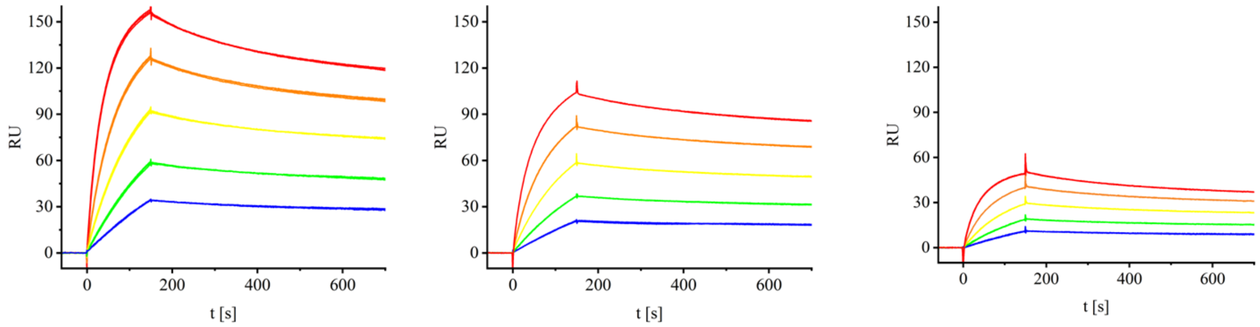

SI Figure 30: Sensorgram of Binding of FITC anti-mouse IgG2b to mouse IgG2b kappa on three PG-PEG biosensorss

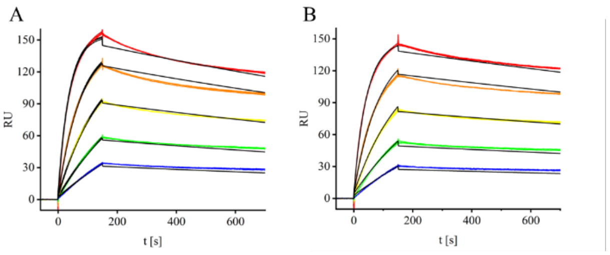

SI Figure 31: Fits of Binding of FITC anti-mouse IgG2b to Mouse IgG2bkappa on a A) PG-PEG biosensor and B) CMD biosensor

Screening of regeneration conditions:

Four different regeneration solutions were injected at 10 µL/min for 30 s (10 mM Glycine-HCl pH = 2; 100 mM HCl; 50 mM NaOH; 4 M $MgCl_2$). Baseline levels were measured immediately after regeneration solution injection. After three startup cycles no loss of RU was observed.

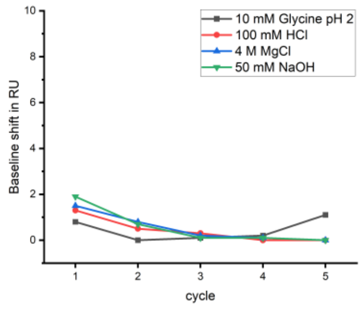

SI Figure 32: Screening of regeneration conditions

SI References

(1) Haag, R.; Sunder, A.; Stumbé, J. F. An approach to glycerol dendrimers and pseudo-dendritic polyglycerols. *J Am Chem Soc* **2000**, *122* (12), 2954-2955..

(2) Sunder, A.; Hanselmann, R.; Frey, H.; Mülhaupt, R. Controlled synthesis of hyperbranched polyglycerols by ring-opening multibranching polymerization. *Macromolecules* **1999**, *32* (13), 4240-4246.

(3) Frey, H.; Haag, R. Dendritic polyglycerol: a new versatile biocompatible-material. *J Biotechnol* **2002**, *90* (3-4), 257-267.

(4) Wallert, M.; Plaschke, J.; Dimde, M.; Ahmadi, V.; Block, S.; Haag, R. Automated Solvent-Free Polymerization of Hyperbranched Polyglycerol with Tailored Molecular Weight by Online Torque Detection. *Macromol Mater Eng* **2021**, *306* (7)

(5) Wolde-Kidan, A.; Herrmann, A.; Prause, A.; Gradzielski, M.; Haag, R.; Block, S.; Netz, R. R. Particle Diffusivity and Free-Energy Profiles in Hydrogels from Time-Resolved Penetration Data. *Biophys J* **2021**, *120* (3), 463-475.
